# Modulation of transcriptional mineralocorticoid receptor activity by casein kinase 2

**DOI:** 10.1038/s41598-017-15418-1

**Published:** 2017-11-10

**Authors:** Stefanie Ruhs, Nicole Strätz, Katja Quarch, Antonia Masch, Mike Schutkowski, Michael Gekle, Claudia Grossmann

**Affiliations:** 1 Julius Bernstein Institute of Physiology, University Halle-Wittenberg, Halle, 06112 Germany; 2 Institute of Biotechnology and Biochemistry, Division of Enzymology, University Halle-Wittenberg, Halle, 06110 Germany

## Abstract

The pathogenesis of cardiovascular diseases is a multifunctional process in which the mineralocorticoid receptor (MR), a ligand-dependent transcription factor, is involved as proven by numerous clinical studies. The development of pathophysiological MR actions depends on the existence of additional factors e.g. inflammatory cytokines and seems to involve posttranslational MR modifications e.g. phosphorylation. Casein kinase 2 (CK2) is a ubiquitously expressed multifunctional serine/threonine kinase that can be activated under inflammatory conditions as the MR. Sequence analysis and inhibitor experiments revealed that CK2 acts as a positive modulator of MR activity by facilitating MR-DNA interaction with subsequent rapid MR degradation. Peptide microarrays and site-directed mutagenesis experiments identified the highly conserved S459 as a functionally relevant CK2 phosphorylation site of the MR. Moreover, MR-CK2 protein-protein interaction mediated by HSP90 was shown by co-immunoprecipitation. During inflammation, cytokine stimulation led to a CK2-dependent increased expression of proinflammatory genes. The additional MR activation by aldosterone during cytokine stimulation augmented CK2-dependent NFκB signaling which enhanced the expression of proinflammatory genes further. Overall, in an inflammatory environment the bidirectional CK2-MR interaction aggravate the existing pathophysiological cellular situation.

## Introduction

The mineralocorticoid receptor (MR) belongs to the nuclear receptor superfamily which includes the progesterone (PR), estrogene (ER), androgene (AR), and glucocorticoid receptor (GR), representing a family of ligand-activated transcription factors (TFs)^1^. In epithelial tissues aldosterone-activated MR mediates sodium and water retention and thereby long term blood pressure regulation. Independently of its hemodynamic effects, inappropriate MR activation promotes pathophysiological effects in the cardiovascular system like inflammation, fibrosis and hypertrophy, leading to endothelial dysfunctions and heart failure^2^. However, the molecular mechanisms involved are incompletely understood.

The human MR contains a regulatory N-terminal AB domain (NTD) followed by a DNA-binding domain C, a hinge region D and the ligand-binding domain EF (LBD)^3^ (Supplemental Figure S1). In the absence of ligands (aldosterone/cortisol), the MR occurs predominantly in the cytosol associated with chaperone molecules like HSP90 that stabilize the MR in a high affinity binding state^3^. Upon ligand binding, MR translocates into the nucleus and binds to glucocorticoid response elements (GRE) as homodimers to modulate gene transcription^4^.

Subsequent to activation of the MR, aldosterone-induced posttranslational modifications (PTMs) like acetylation, oxidation, phosphorylation, sumoylation and ubiquitylation could be detected by an increase in the apparent molecular weight^5^. Incubating aldosterone-stimulated cell lysates with a phosphatase abolished the aldosterone-induced shift^6^ indicating that MR represents a phosphoprotein as has been reported for other steroid hormone receptors (SHR)^1^. Aldosterone-induced MR phosphorylation can modify its binding affinity for hormone response elements (HRE), its nuclear translocation, its interaction with co-regulators^1^ and its ligand binding ability^7^.

Casein kinase 2 (CK2) represents an ubiquitously distributed multifunctional tetra-heteromeric serine (S)/threonine (T) kinase^8^ which is composed of two catalytic (α or α’) and two regulatory (β) subunits and possesses three isoforms: ααββ, αα’ββ, α’α’ββ. CK2β mediates the interaction between the catalytic subunits and recruits CK2 substrates and regulators, modulating substrate selectivity and catalytic activity^9^. CK2 utilizes ATP and GTP as phosphate donors and phosphorylates S/T residues within clusters of acidic amino acids possessing the minimal consensus sequence S*/T*-X-X-D/E. CK2 phosphorylates more than 300 substrates and is involved in many cellular processes like proliferation, apoptosis, differentiation, tumorigenesis and response to cellular stress and DNA damage^10^.

HSP90 is also associated with CK2 and facilitates CK2 activity. Thereby HSP90 enables phosphorylation of CK2 substrates whereas CK2-dependent phosphorylation of HSP90 is required to obtain HSP90 chaperone activity towards client proteins^11,12^. Although CK2 itself does not have molecular chaperone activity, it represents a necessary member of the molecular chaperone system^11^. Thus, it is conceivable that certain HSP90-binding proteins, like the MR, may also be phosphorylated by CK2. Besides, it has been shown that CK2 modulate the genomic activity of other SHR like AR, PR and ER^13^. Formerly, CK2 was described as a constitutively active, non-regulated protein kinase. Recent evidence indicates that CK2 activity and expression are regulated under pathophysiological conditions like inflammation, fibrosis and hypertrophy^9,14,15^ that also facilitate pathophysiological MR activation. Therefore, in this study we investigated i) the influence of CK2 on MR transactivation activity under control conditions, ii) the existence of directly CK2 phosphorylated MR residues, iii) the protein-protein interaction between MR and CK2 and iv) the influence of CK2 on transcriptional MR activity under inflammatory conditions.

## Results

### CK2 influences transcriptional MR activity by affecting posttranslational MR modification and degradation

**Figure 1:**
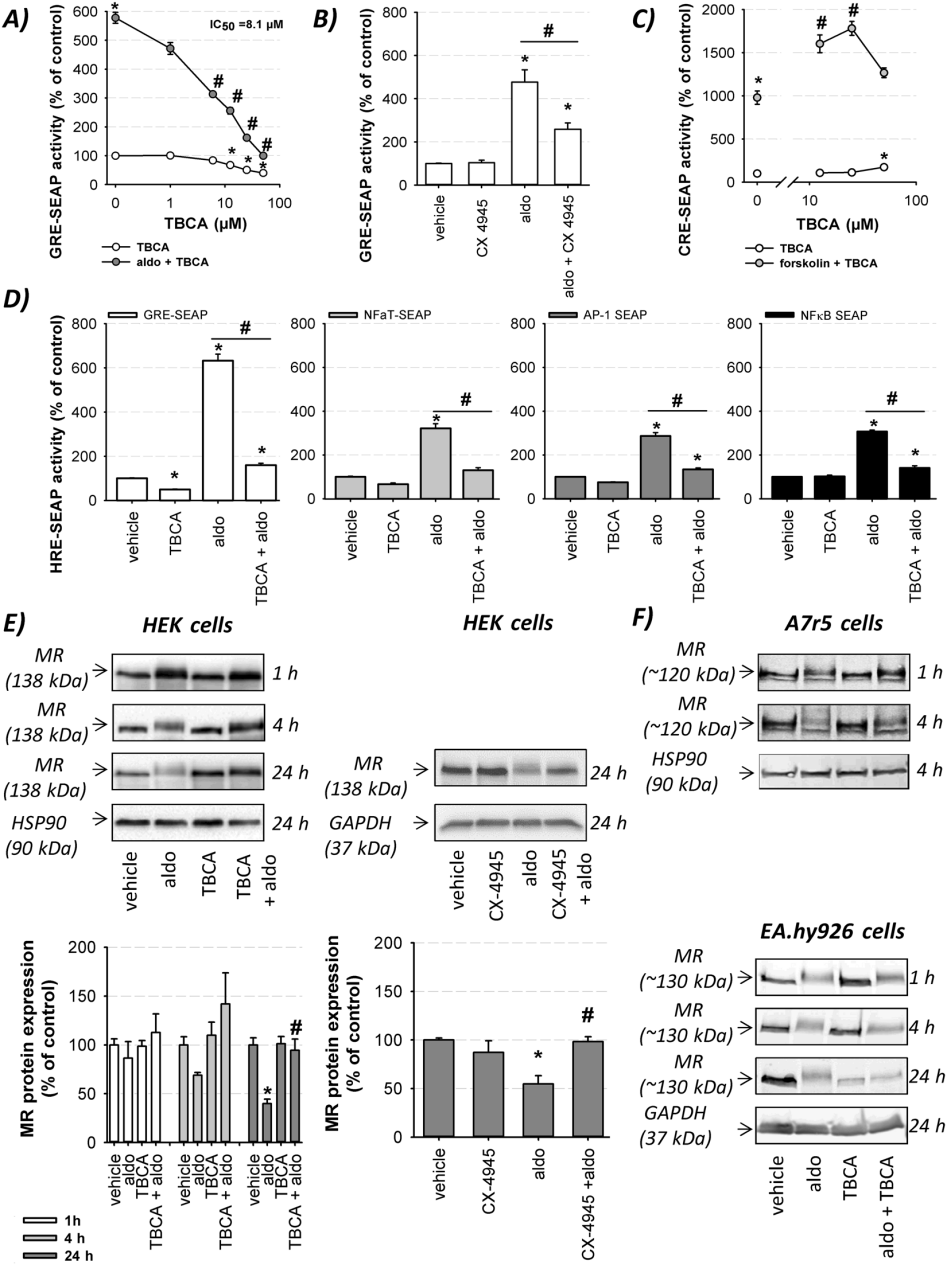
Impact of CK2 inhibition on genomic activity, protein expression and PTM of the MR. (**A**) Increasing TBCA concentrations (1–50 µM) reduced aldosterone (10 nM)-induced genomic MR activity in MR-transfected HEK cells after 24 h as determined by GRE-SEAP reporter gene assay (n = 9–30; N = 3–8; *p ≤ 0.05 vs. control; ^#^p ≤ 0.05 vs. aldo). (**B**) CX-45945 (5 µM) reduced aldosterone (10 nM)-induced genomic MR activity in MR-transfected HEK cells after 24 h as measured by GRE-SEAP reporter gene assay (n = 6–9; N = 3; *p ≤ 0.05 vs. control; ^#^p ≤ 0.05 as indicated). (**C**) Concentration-dependent alteration of forskolin (3 µM)-induced CRE-SEAP activity by TBCA (12.5 to 50 µM) was determined in untransfected HEK cells after 24 h (n = 12–36; N = 4–12; *p ≤ 0.05 vs. control; ^#^p ≤ 0.05 vs. forskolin). (**D**) SEAP reporter gene assays revealed that the aldosterone (10 nM)-activated MR enhanced the transactivation activities of different hormone response elements (GRE-, AP-1-, NFaT- and NFκB SEAP) which were inhibited by TBCA (25 µM) after 24 h (n = 6–9; N = 3; *p ≤ 0.05 vs. control; ^#^p ≤ 0.05 as indicated). (**E**) Western blots of whole MR-HEK cell lysates showed a time-dependent effect of aldosterone (10 nM) ± TBCA (25 µM) or aldosterone (10 nM) ± CX4945 (5 µM) treatment on MR protein expression and PTM of the MR. Representative blots are in the upper panel and the corresponding quantification is depicted in the lower panel (n = 3–10; N = 3–5; *p ≤ 0.05 vs. vehicle; ^#^p ≤ 0.05 vs. aldo). The original unedited Western blots are shown in the supplemental dataset indicated as original Western blots 1. (**F**) Western blots of whole cell lysates showed a time-dependent effect of aldosterone (10 nM) ± TBCA (25 µM) on the endogenously expressed MR and the PTM of the receptor in A7r5 and EA.hy926 cells (n = 2–4; N = 2–4). The original unedited Western blots are shown in the supplemental dataset indicated as original Western blots 2.

To investigate the effect of CK2 phosphorylation on genomic MR activity, a GRE reporter gene assay was employed. The specific CK2 inhibitor TBCA ((2E)−3-(2.3.4.5-tetrabromophenyl)acrylic acid) concentration-dependently diminished basal and aldosterone-induced genomic MR activity after 6 h (Supplemental Figure S2) and 24 h (IC_50_ = 8.1 µM) (Fig. 1A). Additional CK2 inhibitors, TBB (4.5.6.7-Tetrabromo-2-azabenzimidazole) (Supplemental Figure S3) and CX-4945 (3-chloroanilino)benzo[c][2.6]naphthyridine-8-carboxylic acid), confirmed the results and make unspecific effects unlikely (Fig. 1B).

To exclude toxic effects as a cause for reduced transcriptional MR activity, LDH release and protein content were measured but revealed no alterations (Supplemental Table S1). Besides, in the presence of increasing TBCA concentrations, forskolin-induced CRE-SEAP activity was augmented (TBCA: 12.5 µM and 25 µM) or unaltered (TBCA: 50 µM) compared to sole forskolin treatment (Fig. 1C) demonstrating that CK2 does not act as a general and unspecific facilitator of gene expression. Aldosterone-induced activation of NFaT, AP-1 and NFκB SEAP was also inhibited by TBCA (Fig. 1D) revealing that CK2 acts as a positive regulator of MR transactivation activity independently of the analyzed HRE.

MR activation by aldosterone led to an increase in apparent molecular weight of the MR at different time points (Fig. 1E and F) in HEK and endogenous MR-expressing A7r5 and EA.hy926 cells, indicating aldosterone-induced PTMs of the MR which could include different modifications e.g. phosphorylation, ubiquitylation and sumoylation. Additionally, MR activation led to increased receptor degradation compared to controls. TBCA and CX-4945 partially reduced the shift in molecular weight in all three cell lines and MR degradation in HEK and A7r5 cells (Fig. 1E and F) while not affecting MR protein expression under unstimulated conditions. These results suggest that aldosterone-induced PTMs of the MR is induced in part by CK2 and implicated in MR degradation.

### CK2 facilitates DNA-binding of MR and is modulated by the NTD of the receptor

**Figure 2:**
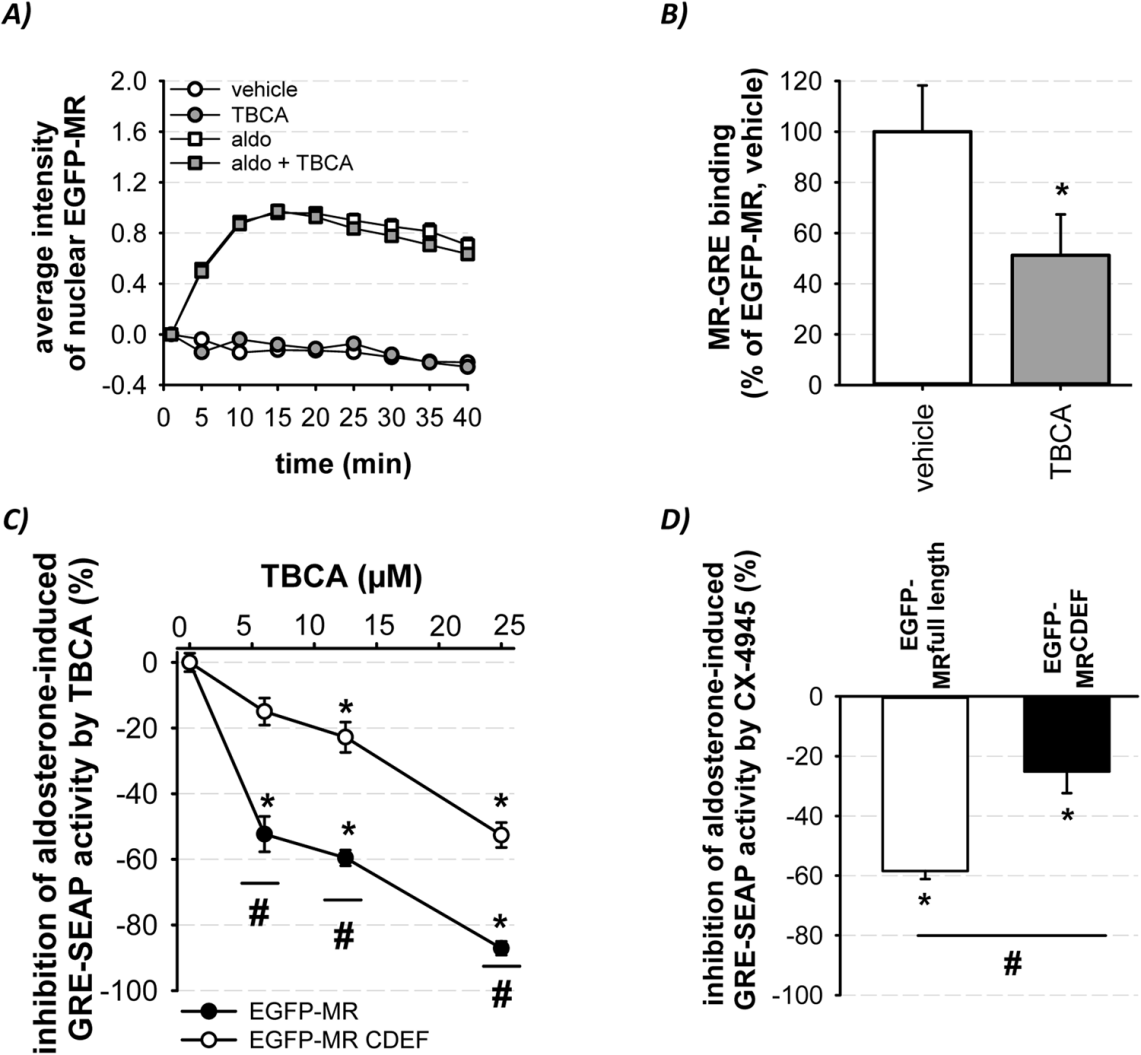
Mechanism of diminished genomic MR activity by CK2 inhibition. (**A**) Cytosolic nuclear EGFP-MR shuttling during aldosterone (10 nM) stimulation was unaffected by TBCA (25 µM) as determined by time lapse experiments (N = 3–4). (**B**) Cytosolic cell extracts from EGFP-MR transfected HEK cells treated with TBCA (25 µM) for 1 h showed a reduced MR-GRE binding compared to controls as determined by Tf-ELISA (n = 8; N = 4; *p ≤ 0.05 vs. vehicle). (**C**) A concentration dependent inhibitory TBCA effect (6–25 µM) on genomic activity of aldosterone (10 nM)-activated EGFP-MR^full length^ and EGFP-MR^CDEF^ was measured by GRE-SEAP reporter gene assay after 24 h (n = 6–18; N = 3–6; *p ≤ 0.05 aldo vs. aldo + TBCA; ^#^p ≤ 0.05 MR^full length^ vs. MR^CDEF^). (**D**) An inhibitory CX-4945 effect (5 µM) on aldosterone (10 nM)-induced GRE-SEAP activity was measured in EGFP-MR^full length^- and EGFP-MR^CDEF^- transfected HEK cells after 24 h (n = 6–9; N = 3; *p ≤ 0.05 aldo vs. aldo + CX-4945; ^#^p ≤ 0.05 MR^full length^ vs. MR^CDEF^).

To investigate the mechanism of TBCA-induced modulation of genomic MR activity, we first investigated whether TBCA treatment altered cytosolic nuclear MR shuttling. Time-lapse experiments showed that TBCA had no impact on nuclear MR translocation (t_0.5_ = 7.5 min; Fig. 2A). Second, the altered MR-DNA interaction after TBCA exposure was analyzed. Cytosolic extracts of EGFP- and EGFP-MR transfected cells, treated with vehicle or TBCA were employed in Tf-ELISA experiments. EGFP-MR binding at DNA (GRE) was clearly higher compared to the negative control EGFP (Supplemental Figure S4) and markedly attenuated by TBCA (Fig. 2B), indicating that CK2 activity facilitates MR-DNA interaction. Third, we analyzed whether the NTD of the MR is necessary for the TBCA-induced inhibition of genomic activity using an AB domain deletion construct (=EGFP-MR^CDEF^). Aldosterone-activated MR^full length^ was more sensitive to inhibition by TBCA than MR^CDEF^ after 6 h (Supplemental Figure S5) and 24 h (Fig. 2C). Likewise, CX-4945 led to more pronounced inhibition of the for MR^full length^ compared to MR^CDEF^ (Fig. 2D). These results indicate that the NTD of the MR has a modulatory effect on CK2-induced regulation of genomic MR activity.

### CK2 phosphorylates MR at S111 and S459 of which S459 shows major relevance for genomic MR activity

**Figure 3:**
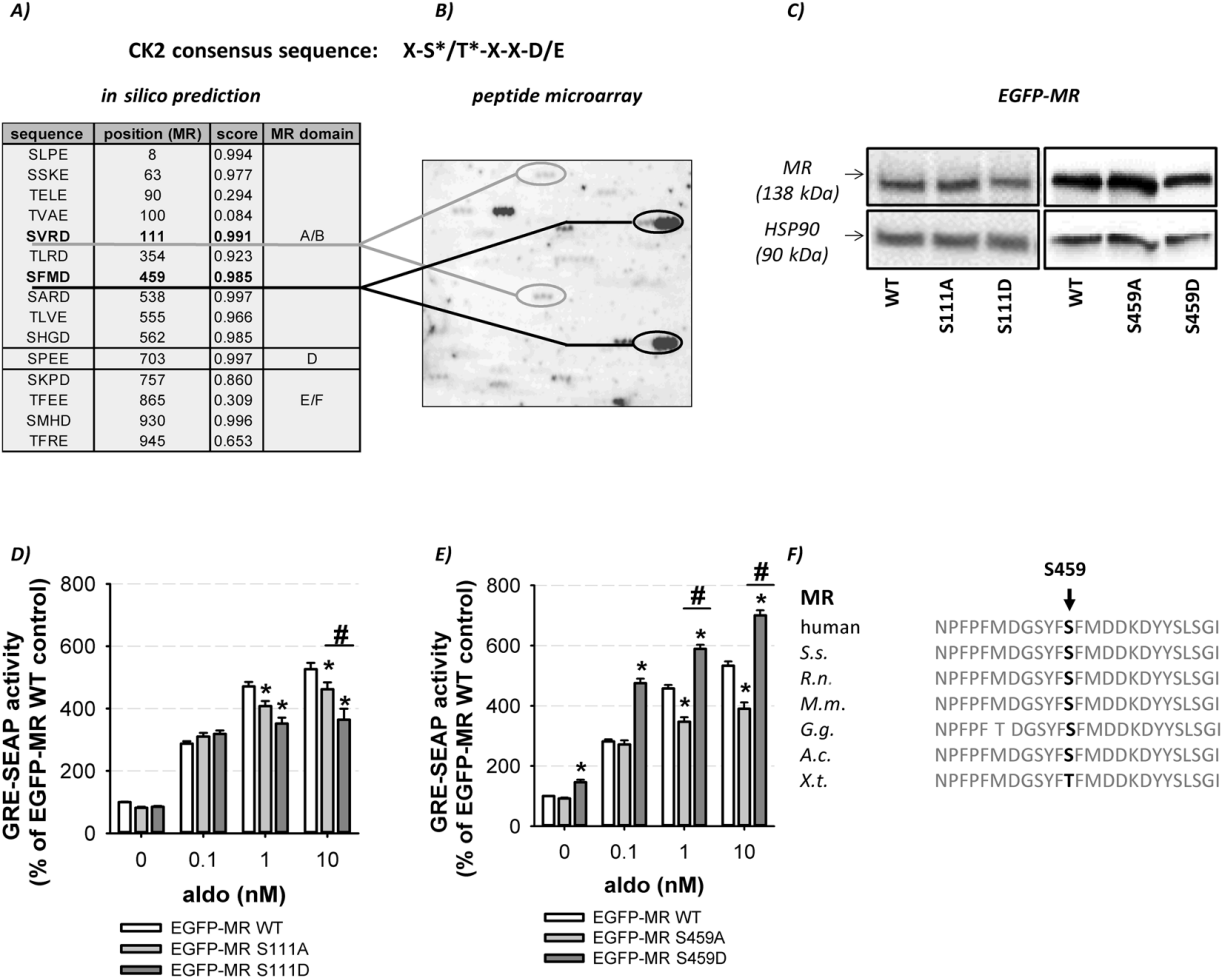
Identification and verification of CK2 phosphorylation sites. (**A**) *In silico* prediction of CK2 consensus sequences were conducted with NetPhos 2.0 (www.cbs.dtu.dk/services/NetPhos/). (**B**) CK2 phosphorylation sites of the MR were identified by peptide microarrays using recombinant CK2 and γ-^33^P-ATP. The incorporated radioactivity was detected by autoradiography after 26 h and identified S111 and S459 as CK2 phosphorylation sites. (**C**) After transfection of HEK cells with WT-MR and mutants (MR S111A/D, MR S459A/D), whole cell lysates were generated. Western blot analysis of WT-MR compared to the MR mutants showed an equal MR protein expression. One representative Western blot of three independent experiments is shown. The unedited original Western blots are shown in the supplemental dataset indicated as original Western blots 3. (**D** and **E**) Genomic MR activities of (**D**) WT-MR, MR S111A and MR S111D and (**E**) WT-MR, MR S459A and MR S459D were determined by GRE-SEAP reporter gene assays after 24 h of aldosterone stimulation (0.1–10 nM). (**D**) MR S111A and MR S111D both showed a reduced regulatory capacity whereas (**E**) MR S459A possessed a reduced and MR S459D an increased regulatory capacity ((**D**): n = 12–24; N = 4–8; (**E**) n = 12–33; N = 4–8; *p ≤ 0.05 vs. vehicle; ^#^p ≤ 0.05 as indicated). (**F**) Conservation of S459 among MR orthologous. S.s., Saimiri sciureus; R.n., Rattus norvegicus; M.m., *Mus musculus*; G.g., *Gallus gallus*; A.c., *Anolis carolinensis*; X.t., *Xenopus tropicalis*.

To identify the exact serine/threonine residue(s) of the MR which are phosphorylated by CK2 we utilized *in silico* prediction by NetPhos2.0 (www.cbs.dtu.dt/services/NetPhos/) (Fig. 3A) and investigated phosphorylation sites by peptide microarrays (Fig. 3B). Synthetic MR peptides with a length of 15 amino acids and an overlap of 12 residues were chemoselectively immobilized on modified glass and *in vitro* phosphorylation reactions were performed using recombinant CK2. Peptides were evaluated according to the following criteria: (i) all nine peptide spots of one MR peptide had to be phosphorylated; (ii) clear signal intensity of peptide spots; (iii) existence of a phosphorylation trend i.e. serine of interest was phosphorylated in more than one overlapping peptide. We identified five MR peptides containing CK2 consensus sequences on the peptide microarray. The two sites with the most consistent results in the *in silico* and *in vitro* analysis, S111 and S459, were analyzed further.

To verify their functional relevance, we converted S111 and S459 by site-directed mutagenesis into alanine (A) or aspartate (D) to acquire phospho-deficient (S111A; S459A) and phospho-mimetic MR mutants (S111D, S459D). Equal protein expression of the MR mutants compared to wild type MR (WT-MR) was verified by Western blot (Fig. 3C). Stimulation of MR S459A with aldosterone led to no alterations in the shift of the molecular weight of the MR compared to WT-MR (Supplemental Figure S6) making it unlikely that this phosphorylation induces further secondary PTMs. Transcriptional activity of the MR mutants was investigated by GRE-SEAP reporter gene assays in comparison to WT-MR. The aldosterone-induced genomic activity of MR S111A and MR S111D were reduced in comparison to WT-MR with the inhibition being most pronounced for MR S111D (Fig. 3D). Since, both MR mutants revealed a general unidirectional change of genomic MR activity, S111 represents a CK2 phosphorylation site with minor relevance for regulating MR GRE transactivation. The same experiments were performed with MR S459A and MR S459D in comparison to WT-MR (Fig. 3E). Basal genomic MR S459D activity was clearly induced compared to WT-MR. Aldosterone-induced MR activity was dose-dependently reduced for MR S459A and elevated for MR S459D (Fig. 3E) suggesting an inverse shift in regulatory capacity. Interestingly, S459 is conserved among orthologues from reptiles to mammals (Fig. 3F), emphasizing its importance. These results indicate that S459 represents a functionally relevant CK2 phosphorylation site which affects aldosterone-induced genomic MR activity.

### MR and CK2 are HSP90-dependently associated in a protein-protein complex

**Figure 4:**
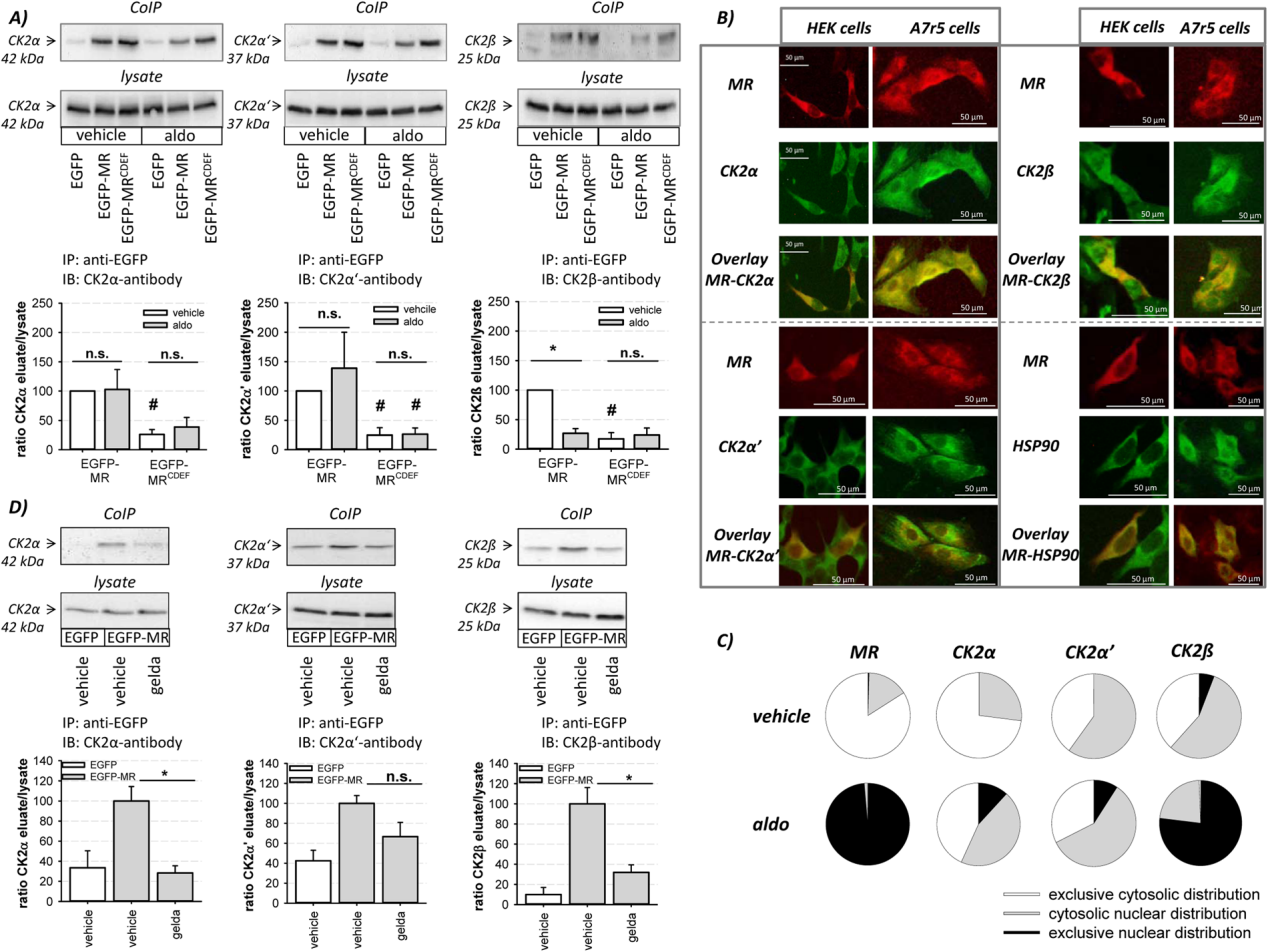
Analysis of MR-CK2 interaction. (**A**) CoIP experiments of EGFP-, EGFP-MR and EGFP-MR^CDEF^ -transfected HEK cells followed by EGFP-MR pulldown and CK2α, CK2α’ and CK2β immunoblotting were performed with whole cell lysates after incubating the cells with aldosterone (10 nM) for 1 h. Quantification of the CoIPs showed that the MR^CDEF^ associates with all three CK2 subunits but that the NTD of the MR is crucial for maximal MR-CK2 interaction (n = 3–6; N = 3–6; *p ≤ 0.05 as indicated; ^#^p ≤ 0.05 EGFP-MR vs. EGFP-MR^CDEF^ vs. equal treatment). The unedited original Western blots are shown in the supplemental dataset indicated as original Western blots 4. (**B**) RFR-MR-transfected HEK and endogenous MR-expressing A7r5 cells were seeded on cover slips. IF staining against MR with Alexa-Fluor 594 (A7r5), CK2α, CK2β, HSP90 with Oregon green and, CK2α’ with Alexa-Fluor 488 were performed. Overlay images indicated a predominantly cytosolic colocalization of the MR with CK2α, CK2β and HSP90 in HEK and A7r5 cells. (**C**) IF staining of RFP-MR-transfected HEK cells against the CK2 subunits showed that aldosterone (10 nM, 1 h) treatment induces an MR-dependent translocation of CK2α and CK2β. (**D**) Western blot analysis of CoIP experiments of EGFP- or EGFP-MR-transfected HEK cells stimulated with vehicle or geldanamycine (2 µM) for 2 h were performed. Quantification of the CoIPs showed an HSP90-mediated MR-CK2α and MR-CK2β interaction (n = 3–9; N = 3; *p ≤ 0.05 as indicated). The unedited original Western blots are shown in the supplemental dataset indicated as original Western blots 5.

CoIP experiments were performed to test whether MR and CK2 assemble in a protein-protein complex. All three CK2 subunits coimmunoprecipitated with the EGFP-MR specifically (Fig. 4A). We obtained related ratios under control conditions for all CK2 subunits (supplemental Figure S7) indicating that no CK2 subunit is preferentially associated with the MR. MR-CK2 co-localization was also verified by IF microscopy. While in HEK cells colocalization of MR with all CK2 subunits was detectable, in endogenously MR-expressing A7r5 cells colocalization of MR was seen for CK2α and CK2β (Fig. 4B; Supplemental Figure S8B,C).

All three CK2 subunits coimmunoprecipitated with the truncated MR^CDEF^ (Fig. 4A). Despite approximately 3 fold higher expression of MR^CDEF^ compared to MR^full length^ (Supplemental Figure S8A), the eluate/lysate ratio of CK2 subunits was lower when immunoprecipitating MR^CDEF^. These results suggest that the CDEF domains by themselves can interact with the CK2 subunits but require the NTD for a maximal efficient interaction. The MR interaction with the catalytic CK2 subunits was unaffected by aldosterone treatment for both receptor variants. In contrast, the MR^full length^-CK2β interaction was strongly reduced by aldosterone to the MR^CDEF^-CK2β interaction level (Fig. 4A). Additionally, IF staining showed that aldosterone-induced MR activation leads to a significantly increased cytosolic-nuclear translocation of CK2α and CK2β subunits (Fig. 4C). These results indicate that aldosterone induces an MR-dependent CK2 translocation.

Geldanamycin, an HSP90 inhibitor, induced no MR degradation within 2 h (Supplemental Figure S8E) but reduced MR-CK2α and MR-CK2β coimmunoprecipitation significantly whereas the MR-CK2α’ interaction was unaffected (Fig. 4D), thus supporting an HSP90-dependent MR-CK2 interaction.

### In an inflammatory environment MR CK2-dependently augments NFκB activity while reducing classical GRE stimulation

An inflammatory environment has been reported to aggravate pathophysiological MR effects and enhance CK2 activity^15,16^. We tested the effect of an inflammatory milieu (cytokine treatment) on MR-CK2 crosstalk and analyzed MR and CK2 mRNA and protein expression and MR activity.

**Figure 5:**
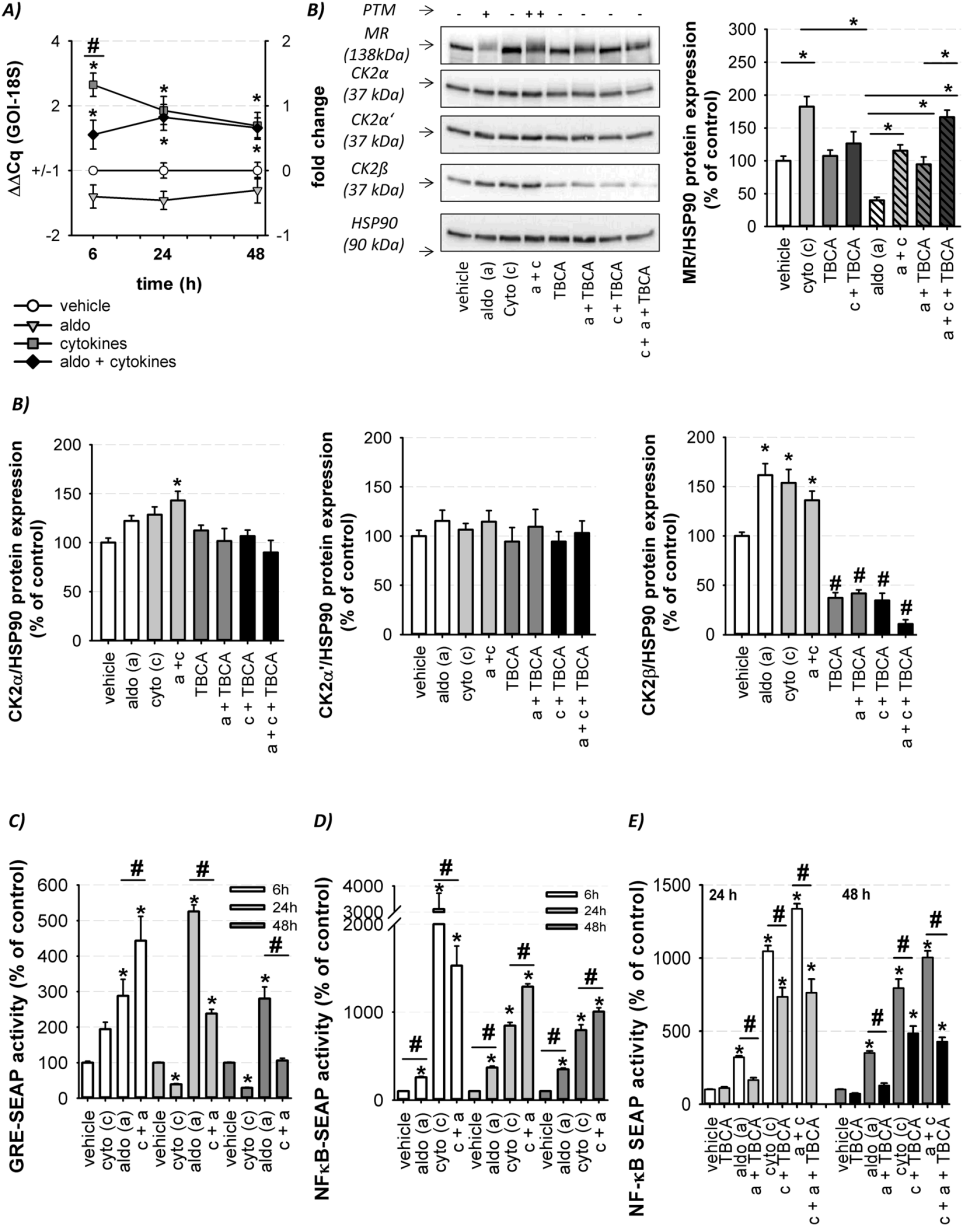
Impact of inflammatory conditions on MR and CK2 expression and genomic MR activity. (**A**) Quantification of MR mRNA alterations after treatment of MR-transfected HEK cells with vehicle, cytokines ± aldosterone (10 nM) for the indicated time points. MR mRNA expression was increased by single cytokine as well as aldosterone + cytokine co-treatment compared to vehicle and aldosterone (n = 9–12; N = 3–4; *p ≤ 0.05 vs. vehicle ^#^ p ≤ 0.05 as indicated). (**B**) MR-transfected HEK cells were treated with vehicle, cytokines, aldosterone (10 nM), TBCA (25 µM) and respective combinations for 24 h followed by MR, CK2α, CK2α’, CK2β and HSP90 immunoblotting as shown by one representive Western. The ratios between the proteins of interest and HSP90 were calculated and revealed an increased MR, CK2α and CK2β protein expression (n = 6–10; N = 4–5; *p ≤ 0.05 vs. vehicle; ^#^p ≤ 0.05 TBCA samples vs. corresponding samples without TBCA). The unedited original Western blots are shown in the supplemental dataset indicated as original Western blots 6. (**C**) Cytokine treatment altered aldosterone (10 nM)-induced GRE-SEAP activity time-dependently in MR-transfected HEK cells (n = 6–18; N = 3–6; *p ≤ 0.05 vs. vehicle; ^#^p ≤ 0.05 as indicated). (**D**) Aldosterone (10 nM) treatment altered cytokine-induced NFκB SEAP activity time-dependently in MR-transfected HEK cells (n = 8–15; N = 4; *p ≤ 0.05 vs. vehicle; ^#^p ≤ 0.05 as indicated). (**E**) The effect of TBCA (25 µM) on aldosterone (10 nM) ± cytokine-induced NFκB SEAP activity was determined after 24 and 48 h in MR-transfected HEK cells (n = 8–15; N = 3–4; *p ≤ 0.05 vs. vehicle; ^#^p ≤ 0.05 as indicated).

Cytokine exposure led to a time dependently increased MR mRNA with maximum after 6 h (Fig. 5A). Western blot analysis confirmed an elevated MR protein expression after 24 h treatment which was CK2 dependent (Fig. 5B). Cytokines and aldosterone treatment elevated the amount of PTM of the MR compared to single aldosterone stimulation which was also CK2-dependent (Fig. 5B). For CK2, an increase in CK2β protein levels was achieved after incubation with cytokines and/or aldosterone. The CK2α protein amount was elevated after aldosterone and cytokine co-treatment. The mRNA levels of CK2α and CK2β were unchanged (Supplemental Figure S9A), suggesting that the treatment furthers CK2α and CK2β protein stability (Fig. 5B). CK2α’ protein level was not affected by any of the incubation conditions (Fig. 5B). These results indicate that an inflammatory milieu increases CK2 protein expression whereby an increased phosphorylation of CK2 targets like the MR becomes possible. Besides, TBCA treatment led to a strong downregulation of CK2β protein expression (Fig. 5B) without affecting CK2β mRNA levels (Supplemental Figure S9B). These results indicate that TBCA inhibits CK2 not only by binding to the ATP-binding site of the catalytic CK2 subunits but also by reducing CK2β protein stability.

To assess the crosstalk between an inflammatory milieu and MR activity we analyzed transcriptional activity of MR in the presence of aldosterone and/or cytokines using a classical GRE- and a NFκB reporter gene assay, known to be responsive to inflammatory stimuli. As expected, aldosterone increased the GRE-reporter activity time dependently with a maximum after 24 h. Cytokines by themselves induced GRE activity after 6 h and strongly reduced GRE activity after 24 and 48 h. Stimulation of cells with aldosterone and cytokines for 6 h resulted in enhanced GRE-SEAP activity while during longer incubation periods cytokines inhibited aldosterone-induced MR activity (Fig. 5C). For the NFκB response element, cytokine treatment led to a strong activation after 6 h that declined during prolonged stimulation (Fig. 5D). Aldosterone treatment induced NFκB reporter activity moderately but suppressed cytokine-induced NFκB activity after 6 h. Prolonged aldosterone incubation increased MR-dependent NFκB reporter activity (Supplemental Figure S10) compared to cytokine treatment alone (Fig. 5D). Overall, the inflammatory milieu leads to a countervailing shift in the activity of these two signaling pathways, MR and NFκB, which could alter gene expression and therefore may be of functional relevance.

Application of TBCA inhibited NFκB activation by aldosterone completely and reduced the cytokine effect partly after 24 and 48 h. The additional NFκB activation by combined incubation of aldosterone and cytokine was completely abolished by TBCA (Fig. 5E). These results suggest that CK2 is involved in cytokine-induced activation of the proinflammatory TF NFκB and also in the additional stimulatory effect of aldosterone that perpetuates an inflammatory environment.

### In an inflammatory environment MR activation elevated the expression of NFκB regulated inflammatory genes

Expression of inflammation-associated genes containing NFκB binding site(s) in their promoter was analyzed time dependently after cytokine and aldosterone stimulation in MR transfected HEK cells (Table 1). As expected, cytokine treatment increased MCP-1, COX-2, EGFR, ICAM-1, VCAM-1 and E-selectin mRNA expression for all indicated time points (Supplemental Figure S11A). Single aldosterone stimulation had no effect but in combination with cytokine treatment led to a further increased mRNA expression compared to cytokine stimulation per se. Additionally, NOX-4 and iNOS mRNA expression, which were not increased during cytokine treatment, were elevated after aldosterone and cytokine co-treatment.

**Table 1:** Influence of aldosterone-stimulated MR activity on cytokine-induced changes in expression of inflammation-associated genes in HEK cells.

aldosterone-intensified cytokine-induced gene expression						
	**cytokine [%] 6 h**	**aldo + cytokine [%] 6 h**	**cytokine [%] 24 h**	**aldo + cytokine [%] 24 h**	**cytokine [%] 48 h**	**aldo + cytokine [%] 48 h**
**MCP-1**	100 ± 2	90 ± 9	100 ± 4	166 ± 18*	100 ± 6	157 ± 16*
**COX-2**	100 ± 10	115 ± 7	100 ± 9	143 ± 11*	100 ± 8	180 ± 32*
**EGFR**	100 ± 5	82 ± 9	100 ± 9	117 ± 16	100 ± 5	134 ± 9*
**ICAM-1**	100 ± 3	115 ± 6	100 ± 9	179 ± 22*	100 ± 6	146 ± 19*
**VCAM-1**	100 ± 5	89 ± 15	100 ± 6	108 ± 4	100 ± 8	174 ± 24*
**E-selectin**	100 ± 5	131 ± 10*	100 ± 5	142 ± 16*	100 ± 6	217 ± 48*
**NOX-4**	100 ± 10	84 ± 14	100 ± 9	167 ± 18*	100 ± 9	124 ± 16
**iNOS**	100 ± 10	132 ± 10	100 ± 9	141 ± 26	100 ± 8	137 ± 11*

Stimulation of MR-transfected HEK cells with cytokines ± aldosterone (10 nM) showed that aldosterone increases mRNA expression of proinflammatory genes in an inflammatory environment for the indicated time points. The cytokine-induced mRNA expression of proinflammatory genes was normalized to 100% compared to the effect of cytokines plus aldosterone (n = 9–12; N = 3–4; *p ≤ 0.05 vs. cytokine).

The influence of an aldosterone-intensified inflammatory environment was analyzed in human endothelial cells (TIMEs), a cell model with closer proximity to pathophysiological vascular MR effects. Analogous to the results obtained by HEK cells, we observed an induction of the mRNA levels of MCP-1, COX-2, EGFR, VCAM-1, E-selectin and NOX-4 by cytokine treatment (Supplemental Figure S11B) which was augmented by aldosterone co-treatment (Table 2). These results confirm the aggravating effect of MR activation on the expression of cytokine-regulated genes involved in the inflammatory response.

## Discussion

Clinical trials demonstrate that the MR is involved in the development of cardiovascular diseases^2^ but the underlying mechanisms are incompletely understood. We focused on the analysis of MR phosphorylation, which appears to play a major role in modulating intrinsic MR function e.g. by regulating ligand binding^6,7^. In silico analyses predict CK2 as a promising candidate for MR phosphorylation due to several CK2 consensus sequences in the NTD, hinge region and LBD (Fig. 3A). However, until now no evidence exists for CK2-induced alterations of MR functions.

For the first time, we show that pharmacological CK2 inhibition reduced MR transactivation activity (Fig. 1A and D) indicating that CK2 represents a MR co-activator. Genomic AR and ERα activities are also sensitive to CK2 inhibition indicating that these SHRs are regulated by CK2, too^13,17^. A general unspecific inhibitory transcriptional TBCA effect was excluded because forskolin-induced CRE-SEAP activity was increased by TBCA co-treatment (Fig. 1C) indicating that CK2 acts as a co-repressor of CREB function. Analyses of the D. melanogaster homolog dCREB showed that the phosphorylation of conserved CK2 sites in dCREB inhibits DNA binding^18^. Unfortunately, CK2α/CK2α’ downregulation by siRNA was not sufficient to confirm our former results due to incomplete knock down of the CK2 subunits.

Our further studies focused on understanding the potential mechanisms involved in CK2-induced modulation of transcriptional MR activity. Our investigations reveal a direct CK2-induced MR phosphorylation of S111 and S459 (Fig. 3B) as has been described for PR-B^19^. The functional relevance of the highly conserved S459 was confirmed by site-directed mutagenesis (Fig. 3D and E). CK2 phosphorylation at S459 augmented transcriptional MR activity to a similar degree as has been described for PR^20^.

Mechanistically, we identify a CK2-independent nuclear MR shuttling (Fig. 2A) in contrast to the AR^13^ but we could show a CK2-dependent increase in MR-DNA interaction (Fig. 2B) as has been reported for ERα^21^. Preliminary Tf-ELISA results suggest that the MR S459A binding to DNA was reduced compared to MR WT. These results support our hypothesis that phosphorylation of S459 facilitates MR-DNA interaction due to conformational changes of the receptor and/or by MR co-activator binding whereby genomic MR activity is enhanced.

The NTD of the MR, a highly disordered region, consists of 3 subdomains: activating function AF1a (1–163), inhibitory domain and AF1b (445–602) with serval putative phosphorylation sites. S459 is located in the NTD within AF1b which mediates the interaction with the transcriptional apparatus^3^ and represents a region of co-regulator binding (e.g. ELL)^22^. PTM of residues especially in the NTD of the MR can alter the receptor DNA binding ability due to conformational changes or altered cofactor recruitment. Besides, in AF1b (450–460) exists a highly conserved binding motif for the scaffolding protein caveolin which enables a crosstalk between SHR and other signaling pathways^23^.

Additionally, the change in genomic MR activity induced by mutation of S459 was smaller than the pharmacological CK2 inhibition, indicating that CK2 can phosphorylate additional S/T residues i) of the MR in its CDEF domain (Fig. 3A) and/or (ii) MR co-regulators directly. For other SHRs like ERβ and GR it has been established that SHR co-regulators are phosphorylated by CK2 and thereby modulating transcriptional SHR activity^24,25^. So far, it is not known whether i) CK2-induced phosphorylation of MR-S459 alters the interaction with MR co-regulators, ii) MR-caveolin signaling and/or iii) whether known MR co-activators are regulated by CK2-induced phosphorylation directly which will be investigated in further experiments.

Direct CK2-induced MR phosphorylation implies that both proteins are associated in a protein-protein complex directly or HSP90 mediated as described for MR-PP2BAβ^26^. In HEK and the endogenously MR-expressing A7r5 cells, IF microscopy and CoIP experiments reveal that the MR interacts with the catalytic CK2α and regulatory CK2β subunits (Fig. 4A and B) indicating that the MR is associated with either a tetrameric CK2 configuration or with free CK2 subunits. New studies reveal that the CK2 tetramer is not a stable but a dynamic, transient heterocomplex making it likely that the individual CK2 subunits perform tetramer-dependent and tetramer-independent functions^27^. This raises the possibility that the MR may be phosphorylated directly by the tetrameric CK2 form (α_2-_β_2_) in the cytosol or by MR-CK2α/α’ complexes during cytosolic nuclear MR shuttling since aldosterone treatment reduces MR-CK2β interaction (Fig. 4A). For the MR-CK2 interaction the CDEF domains of the MR are sufficient but to obtain a maximal CK2-MR interaction the NTD is crucial (Fig. 4A). Until now only a few MR co-regulators are established which bind either in the NTD via an “undescribed” binding motif ^22^ or in the LDB of the MR via a L-xx-LL motif ^3^. Protein sequence analysis reveal that CK2α and α’ in contrast to CK2β dispose of a L-xx-LL motif so that a direct interaction with the LBD of the MR seems possible. Conversely, inhibition of HSP90 diminished MR-CK2α and MR CK2β but not CK2α’coimmunoprecipitation (Fig. 4D). These results suggest that CK2α’ could probably interact directly with the MR in its CDEF (LBD) domains in a cell context specific manner whereas the MR-CK2α and MR-CK2β interaction is HSP90 mediated. The existence of the NTD of the MR fostered the MR-CK2 interaction probably via the help of HSP90.

Numerous studies show that the damaging effects of aldosterone in the cardiovascular system depend on cardiovascular risk factors e.g. high salt intake^28^, hypertension^29^ and obesity^30^ which generate a cellular milieu characterized by reduced antioxidative capacity, elevated oxidative stress^31^ and chronically increased cytokine levels^32^.

We found that cytokine treatment elevates MR mRNA and protein expression and also increases the amount of PTM of the MR after aldosterone co-treatment (Fig. 5A and B). Formerly, CK2 was regarded as a constitutively active kinase but recent data indicate that CK2 activity and protein expression is enhanced by proinflammatory/profibrotic cytokines^33,34^, hypertrophic agents^14^ and during myocardial infarction^35^. We also observed an increased CK2β protein expression during cytokine treatment (Fig. 5A). CK2 has been found to phosphorylate and thereby modulate the actions of several TFs, including NFκB, STAT-1, SP-1 and AP-1 that are key players of inflammation-associated cellular and tissue changes^36^. Consequently, CK2 is implicated in the generation of pathophysiological processes^14,34,37^ e.g. inflammation^15^ and is activated under related pathophysiological conditions like the MR.

Unexpectedly, aldosterone-induced genomic MR activity at GRE was long-term reduced during co-exposure to an inflammatory environment (Fig. 5C). In contrast, we observed a moderate aldosterone MR-induced induction of the NFκB signaling as described in literature but which was unable to alter inflammation-associated gene expression. During aldosterone-induced MR activation the receptor translocates into the nucleus by its chaperones HSP90 and FKBP52^38^. Erlejman *et al*. showed that the genomic NFκB activity is strongly enhanced by increasing nuclear amounts of FKBP52^39^. Consequently, we hypothesize that MR activation and nuclear shuttling can enhance genomic NFκB activity by elevating the amount of nuclear FKBP52. Aldosterone-induced NFκB activity was prevented by CK2 inhibition (Fig. 5E) as previously shown for MR antagonists^40^.

Besides, proinflammatory cytokine treatment strongly elevated NFκB activity which was partly mediated by CK2 (Fig. 5D and E). Several investigations show that CK2 is a component of the cytokine-induced NFκB signaling pathway. It can phosphorylate the NFκB inhibitor IkB followed by IkB degradation and elevated nuclear NFκB translocation. Furthermore, CK2 phosphorylation of the NFκB subunit p65 results in a more efficient formation of enhanceosomes by forming docking sites for co-activators^41^. The coincidence of cytokines and aldosterone treatment augmented additively long-term cytokine-induced NFκB activity compared to single cytokine stimulation in a CK2-dependent manner (Fig. 5D and E) and elevated the expression of inflammation-associated genes in HEK and endogenously MR-expressing TIME cells (Tables 1 and 2). These results reveal that aldosterone per se is not capable of eliciting damage but can aggravate pre-existing inflammatory changes and thereby lead to pathophysiological MR effects^28^

**Table 2:** Influence of aldosterone-stimulated MR activity on cytokine-induced changes in expression of inflammation-associated genes in TIME cells.

aldosterone-intensified cytokine-induced gene expression		
	cytokine [%] 24 h	aldo + cytokine [%] 24 h
MCP-1	100 ± 5	158 ± 19*
**COX-2**	100 ± 9	134 ± 15*
**EGFR**	100 ± 6	145 ± 13*
**ICAM-1**	100 ± 8	106 ± 13
**VCAM-1**	100 ± 8	177 ± 15*
**E-selectin**	100 ± 9	165 ± 14*
**NOX-2**	100 ± 10	196 ± 63
**NOX-4**	100 ± 9	161 ± 17*
**eNOS**	100 ± 5	115 ± 98

Stimulation of endothelial cells with cytokines ± aldosterone (10 nM) showed that aldosterone increases mRNA expression of proinflammatory genes in an inflammatory environment after 24 h stimulation. The cytokine-induced mRNA expression of proinflammatory genes was normalized to 100% compared to the effect of cytokines plus aldosterone (n = 12; N = 4; *p ≤ 0.05 vs. cytokine).

**Figure 6:**
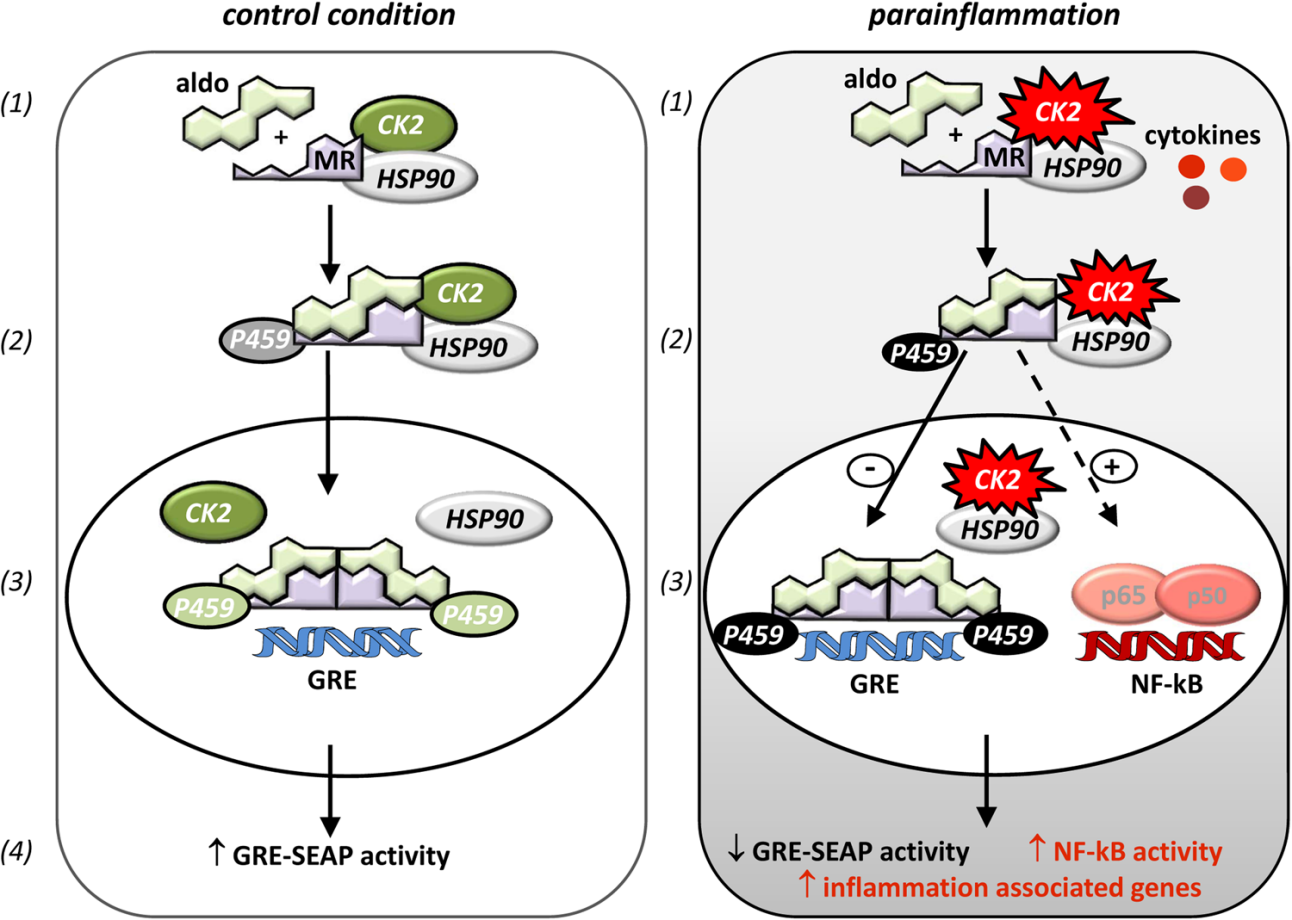
Model: Influence of CK2 on genomic MR activity. **Control condition:** CK2 represents a positive regulator of genomic MR activity under control conditions: (1) CK2 and MR are associated in a protein-protein complex which is mediated by HSP90. (2) CK2 directly phosphorylates the MR on S459. (3) CK2 increase MR-DNA (GRE) interaction/binding. (4) CK2-induced MR phosphorylation increases genomic MR activity. **Parainflammation:** MR activation aggravates an existing inflammatory situation through CK2 dependent mechanisms: (1) An inflammatory environment elevates the expression level of CK2α, CK2β and MR. This leads to an increased amount of posttranslationally modified MR after aldosterone treatment through CK2-mediated phosphorylation. (2) The cytokines alter the temporal pattern of MR activity and reduced GRE activity. In contrast, MR activation extends the temporal pattern of cytokines and increases NFκB activity. (3) In an inflammatory environment additional MR activation aggravates the pathophysiological situation by enhancing the expression of inflammation-associated genes.

Overall, these results indicate that CK2 represents a co-activator of genomic MR activity by i) direct MR phosphorylation e.g. on S459 and ii) probably by indirectly phosphorylation of MR co-regulators under control conditions. Additionally, activated MR aggravates an existing inflammatory situation via CK2-dependent mechanisms by augmenting NFκB activity and consequently the expression of proinflammatory genes (Figure 6).

## Methods

### Cell Culture

HEK-293 cells, A7r5, EA.hy926 and TIME cells (American Type Culture Collection) were cultivated in the recommend media as indicated in Supplemental Table S2 and cultivated 37 °C with 5% CO_2_. 24 h prior to the experiments, cells were cultivated in serum-free medium for 24 h and treated with vehicle (DMSO 0.1%), aldosterone or a cytokine mixture composed of IL-1β (10 ng/ml), IL-6 (20 ng/ml), TNFα (20 ng/ml). Single exception represents the TIMEs which made quiescent by utilization of charcoal-stripped steroid-free medium with a reduced FCS content (2%) for 24 h.

### Transfection

Transfection of HEK cells with MR expression vector pEGFP-C1-MR (kind gift of N. Farman, Paris), pEGFP-MR^CDEF^ ^42^, pEGFP-C1 (Clontech), pMR-DsRed2-N1 (RFP)-MR^26^ or pcDNA3.1-His-LacZ (control vector, Invitrogen) was performed as described before^42^ with Polyfect reagent (Qiagen).

### Western Blot

Cells were lysed with RIPA buffer (Supplemental Table S3A) and protein content was determined by BCA assay. 50–75 µg protein was separated by 10–12% SDS-PAGE, transferred to nitrocellulose membrane and incubated with primary and secondary antibodies as listed in Supplemental Table S3B. Protein bands were detected in the linear range with the ECL system (Amersham Corp.). Densitometry analyses were performed with Quantity One^®^ (Biorad).

### Coimmunoprecipitation (CoIP)

Cells were transfected with pEGFP-C1, pEGFP-hMR, pEGFP-hMR^CDEF^ and incubated with vehicle, aldosterone (10 nM) for 1 h or geldanamycin (2 µM) for 2 h. CoIPs were performed with anti-GFP-coupled magnetic beads and µ columns from Miltenyi (Germany) as recommended by the manufacturer followed by Western blot analysis.

Due to a stronger EGFP-MR^CDEF^ protein expression compared to MR^full length^ (MR^CDEF^/MR^full length^ supplemental Figure S8A) we calculated the CoIP experiments as follows:

1 $$\frac{({({\rm{CK2}}\exp {\rm{.}}{\rm{of}}{{\rm{MR}}}^{{\rm{full}}{\rm{length}}})}_{{\rm{eluate}}}-{({\rm{CK2}}\exp .{\rm{of}}{\rm{EGFP}})}_{{\rm{eluate}}})/}{{({\rm{CK2}}\exp .{\rm{of}}{{\rm{MR}}}^{{\rm{full}}{\rm{length}}})}_{{\rm{lysate}}}}$$

2 $$\frac{({({\rm{CK2}}\exp .{\rm{of}}{{\rm{MR}}}^{{\rm{CDEF}}})}_{{\rm{eluate}}}-{({\rm{CK2}}{\rm{expression}}{\rm{of}}{\rm{EGFP}})}_{{\rm{eluate}}})/}{({{\rm{MR}}}^{{\rm{CDEF}}}{/\mathrm{MR}}^{{\rm{full}}{\rm{length}}}\,{\rm{coefficient}}\ast {({\rm{CK2}}{\rm{expression}}{\rm{of}}{{\rm{MR}}}^{{\rm{CDEF}}})}_{{\rm{lysate}}})}$$

### Immunofluorescence (IF) analysis

For IF analysis RFP-MR transfected HEK and endogenous MR expressing A7r5 cells were cultivated on 10 well glass slides. The experimental procedure was performed as described earlier^26^. The utilized antibodies are listed in Supplemental Table S4.

### SEAP (secreted alkaline phosphatase) reporter gene assay

Transactivation of EGFP-MR and EGFP-MR mutants was assessed by the Mercury Pathway Profiling reporter gene assay system (Clontech) using secretory alkaline phosphatase (SEAP) as reporter^42^. Cells were cotransfected with pGRE-SEAP, pNFaT-SEAP, pAP-1-SEAP, pNFκB-SEAP and pEGFP-hMR constructs or pCRE-SEAP as indicated. SEAP activity in the media was determined with the AttoPhos System (Promega) and normalized to the transfection control β-galactosidase.

### Time lapse experiments

Time lapse experiments were performed with a digital BZ-8000 fluorescence microscope from Keyence (Osaka, Japan) with an incubation chamber as described earlier^4^. The nuclear-cytoplasmic distribution after incubation with vehicle or TBCA (25 µM) ± aldosterone (10 nM) was monitored for 40 minutes and evaluated with ImageJ software (National Institutes of Health, USA).

### ELISA-based transcription factor DNA binding assay (Tf-ELISA)

Cytosolic extract of EGFP or EGFP-MR transfected HEK cells which were treated with vehicle or TBCA (25 µM) ± aldosterone (10 nM) for 1 h were isolated with the Nuclear Extract Kit (Active Motif) as recommended by the manufacturer. Protein content was determined using Bradford reagent (Biorad). For Tf-ELISA experiments biotinylated DNA-(GRE) probes are captured in streptavidin-coated wells and the TFs bound were detected by specific antibodies as described earlier^42^.

### Peptide microarrays

Peptide microarrays were produced by JPT peptide Technologies GmbH (Berlin, Germany). Every MR peptide was composed of 15 aa with 12 aa overlap resulting in 341 peptides. Each peptide was spotted three times in triplicates resulting in 9-fold presentation of each individual peptide.

Microarrays were incubated with recombinant CK2 (500 U) (New England Biolabs, USA) in a kinase buffer (in mM: Tris-HCl 20, pH 7.5, MgCl_2_ 10, KCl 50, ATP 0.25 µM, 0.36 MBq γ-^33^P-ATP) for 2 h at 25 °C in a humid chamber as described by^43^. The incorporated radioactivity was detected after rigorous washing by exposure of the peptide microarrays for 26 h to imaging plates (Fuji BAS-MS, Fuji Photo Film Co.) followed by readout with a FLA 3000 phosphor imager (Fuji). Control experiments with radioisotopically labeled ATP in buffer without kinase did not yield any signal after phosphor imaging.

### Site-directed mutagenesis experiments

The EGFP-MR mutants S111A, S111D, S459A and S459D were prepared using the MR expression vector pEGFP-C1-hMR and the QuikChange II XL Site-Directed Mutagenesis Kit (Agilent Technologies, Santa Clara, USA) as recommended by the manufacturer. Cloning results were confirmed by sequencing (Eurofins MWG Operon).

### Quantitative PCR (qPCR)

For mRNA expression analysis HEK and TIME cells were incubated with vehicle, aldosterone (10 nM) ± cytokines: IL-1β, IL-6, TNFα for the indicated time points (Fig. 6A and B). Total RNA was isolated using the InviTrap Spin Tissue RNA Mini Kit (Stratec Biomedical) with an additional DNase I digestion. SuperScript II reverse transcriptase (Invitrogen) and random primers were used for reverse transcription. Real time amplification was performed using Platinum SYBER Green qPCR Supermix (Invitrogen). Fold changes in expression levels were calculated by the 2^∆∆Cq^ method, using the 18 S signal for normalization. Details of the utilized primers are listed in Supplemental Table S5.

### Statistics

Data are presented as mean ± standard error mean (SEM). Significance of difference was tested by unpaired Student’s t-test or One-way ANOVA with p ≤ 0.05 considered statistically significant. N represents the number of individual experiments and n the number of wells or culture dishes investigated per experiment.

## Electronic supplementary material

Supplementary dataset
