# Supplementary material for: Modulation of transcriptional mineralocorticoid receptor activity by casein kinase 2
Source: Sci Rep. 2017 Nov 10;7:15340. doi: 10.1038/s41598-017-15418-1 (PMC5681688; doi:10.1038/s41598-017-15418-1)
Supplement: Supplementary file 1 — Supplementary dataset [file 41598_2017_15418_MOESM1_ESM.doc]

**Supplementary data**

**Title:**

**Modulation of transcriptional mineralocorticoid receptor activity by casein kinase 2**

**Authors:** Stefanie Ruhs*^1^, Nicole Strätz^1^, Katja Quarch^1^, Antonia Masch^2^, Mike Schutkowski^2^, Michael Gekle^1^ and Claudia Grossmann^1^

^1^Julius Bernstein Institute of Physiology, University Halle-Wittenberg, Halle 06112, Germany

^2^ Institute of Biotechnology and Biochemistry, Division of Enzymology, University Halle-Wittenberg, Halle 06110, Germany

Address of correspondence: Stefanie Ruhs, Julius Bernstein Institute of Physiology, University Halle-Wittenberg, Magdeburger Straße 6, 06112 Halle (Saale), Germany, Tel.: ++49 345 557 4434; Fax: ++49 345 557 4019; E-mail: [stefanie.ruhs@medizin.uni-halle.de](mailto:stefanie.ruhs@medizin.uni-halle.de)

**Supplemental data**

**Figure S1, related to Introduction**

**Supplemental Figure S1: Schematic representation of the MR domains.**

The human MR contains 984 amino acids and is composed of an N-terminal AB domain (NTD), a DNA-binding domain C, a short hinge region D and a ligand binding domain (LBD) EF. The NTD is subdivided into the activation functions AF-1a (1-163) and AF-1b (438-602) and an inhibitory domain (ID, 164-437) in between. The utilized MR full length construct is consists of all MR domains (A-F). The truncated version MR CDEF lacks the regulatory AB domain.

**Table S1, related to Figure 1**

**A)**

**
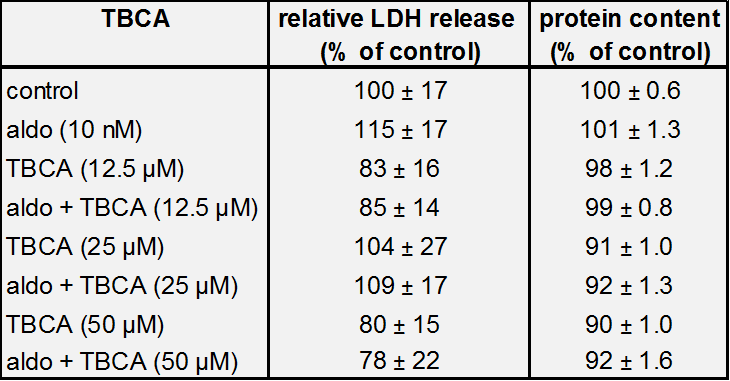
**

**B)**

**
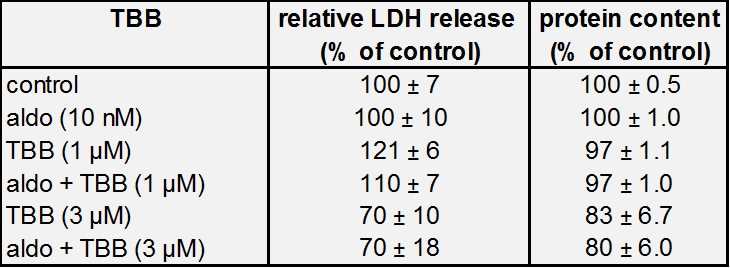
**

**Supplemental Table S1: Influence of CK2 inhibitors TBCA and TBB on cellular viability.**

Cellular viability was unchanged during stimulation of MR-transfected HEK cells with increasing **(A)** TBCA concentrations ± aldosterone (10 nM) or **(B)** TBB concentrations ± aldosterone (10 nM) measured by of LDH release as described eralier (Ruhs et al., 2012) and cellular protein content after 24 h.

(LDH release: n = 9-18; N = 3-6; BCA: n = 9-52; N = 3-17).

**Figure S2, related to Figure 1A**

**Supplemental Figure S2: Influence of TBCA on aldosterone-induced GRE-SEAP activity after 6 h.**

TBCA concentration dependently reduced aldosterone (10nM)-induced genomic MR activity in MR-transfected HEK cells after 6 h as measured by GRE-SEAP reporter gene assay (n = 6-12; N = 2-4; *p ≤ 0.05 vs. control; #p ≤ 0.05 vs. aldosterone).

**Figure S3, related to Figure 1A-B**

**
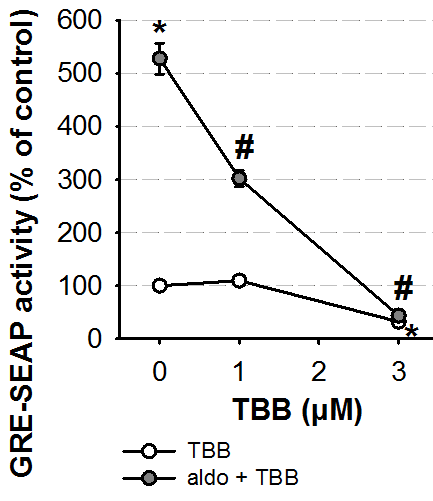
**

**Supplemental Figure S3: Influence of TBB on aldosterone-induced GRE-SEAP activity.**

TBB (1 and 3 µM) concentration dependently reduced basal and aldosterone (10nM)-induced genomic MR activity in MR-transfected HEK cells after 24 h as measured by GRE-SEAP reporter gene assay (n = 12-36; N = 4-12; *p ≤ 0.05 vs. control; #p ≤ 0.05 vs. aldosterone).

**Figure S4, related to Figure 2B**

**
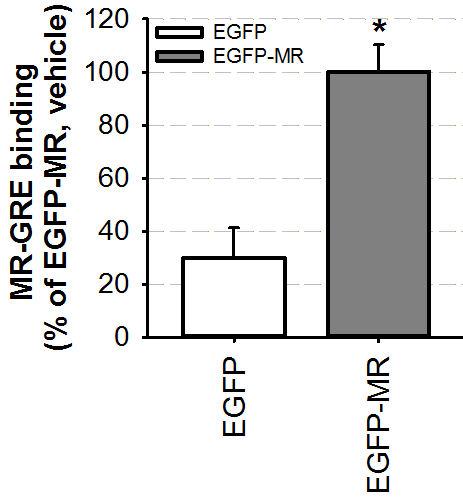
**

**Supplemental Figure S4: Verification of the MR binding at GRE compared to negative control EGFP**

EGFP or EGFP-MR transfected HEK cells were incubated with vehicle for 1 h followed by the generation of cytosolic extracts. Cytosolic extracts of EGFP-MR transfected HEK cells showed a clearly higher GRE binding (3.3 fold) as the EGFP transfected HEK cells (negative control) (n =4-8; N = 4; *p ≤ 0.05 vs. vehicle).

**Figure S5, related to Figure 2C**

**Supplemental Figure S5: Comparison of the TBCA-induced inhibition of genomic MR^full length^ versus MR^CDEF^ activity after 6h stimulation.**

A concentration dependent inhibitory TBCA effect (12.5-25 µM) on genomic activity of aldosterone (10nM)-activated EGFP-MR^full length^ and EGFP-MR^CDEF^ was measured by GRE-SEAP reporter gene assay after 6 h (n=4-12; N=2-4; *p ≤ 0.05 vehicle vs. aldo; #p ≤ 0.05 MR^full length^ vs. MR^CDEF^).

**Figure S6, related to Figure 3C**

**
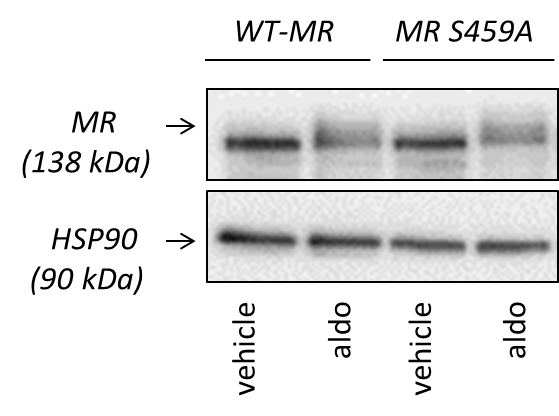
**

**Supplemental Figure S6: Analysis of the aldosterone-induced shift of the molecular weight of WT-MR compared to MR S459A.**

WT-MR and MR_S459A transfected HEK cells were incubated with vehicle (DMSO 0.1%) or aldosterone 10 nM for 1 h followed by whole cell lysate generation. Western blot analysis showed that the aldosterone-induced shift of the molecular weight of the MR was comparable between WT-MR and MR S459A. (n =4; N = 2).

**Figure S7, related to Figure 4**

**
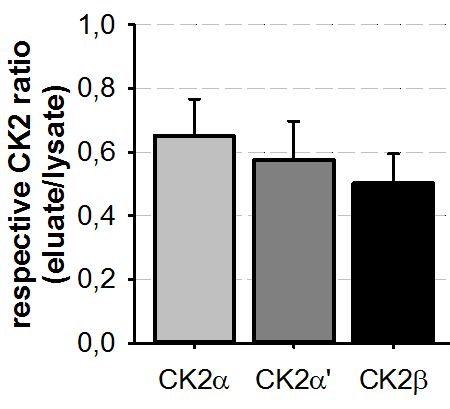
**

**Figure S7: Calculation of the ratios (eluate/lysate) of the respective CK2 subunits after CoIP.**

CoIP experiments of EGFP-MR transfected HEK cells followed by EGFP-MR pulldown and CK2α, CK2α’ and CK2β immunoblotting were performed with whole cell lysate after incubating the cells with vehicle (DMSO 0.1%) for 1h. Ratios between protein contents of CoIP eluates and corresponding cell lysates were calculated and reveal that no CK2 subunit is preferentially associated with the MR (n=16-20; N=8-11).

**Figure S8, related to Figure 4**

**A)**

**
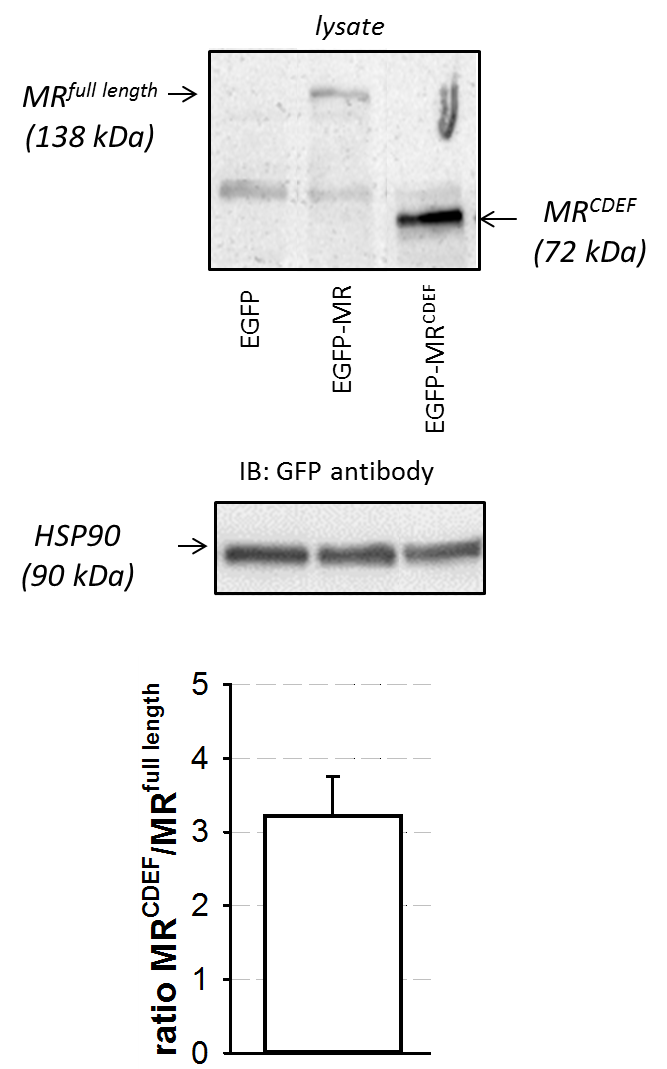
**

**Figure S8A: EGFP-MR^full length^ and EGFP-MR^CDEF^ show different protein expression levels in HEK cells.**

Cell lysates of vehicle treated EGFP-MR^full length^ and EGFP-MR^CDEF^ transfected HEK cells were generated followed by GFP immunoblotting. The ratio of EGFP-MR^CDEF^ to EGFP-MR^full length^ protein expression was calculated and show a 3 fold increased EGFP-MR^CDEF^ expression compared to EGFP-MR^full length^ (n=6; N=6).

**B)**

**
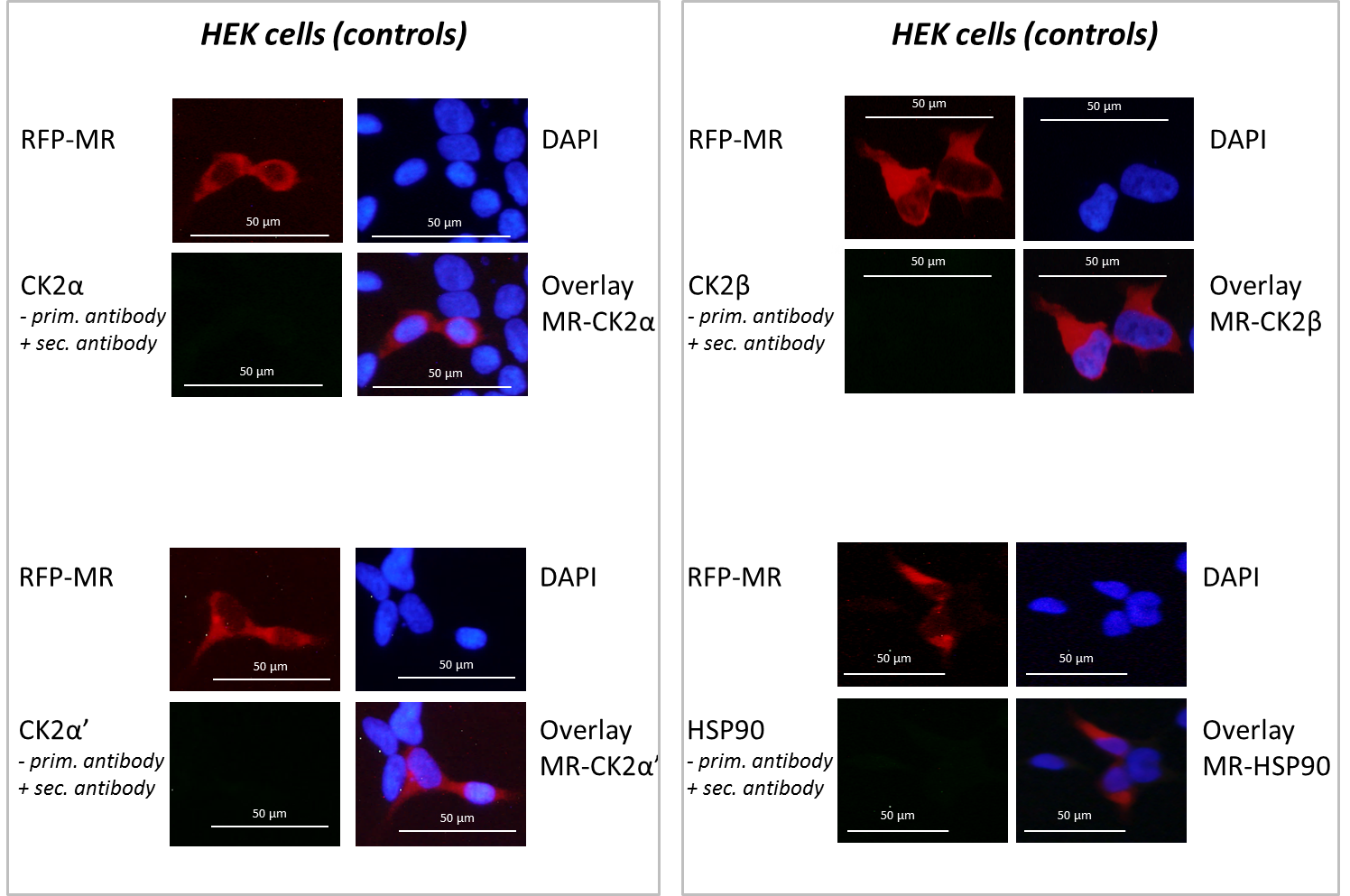
**

**C)
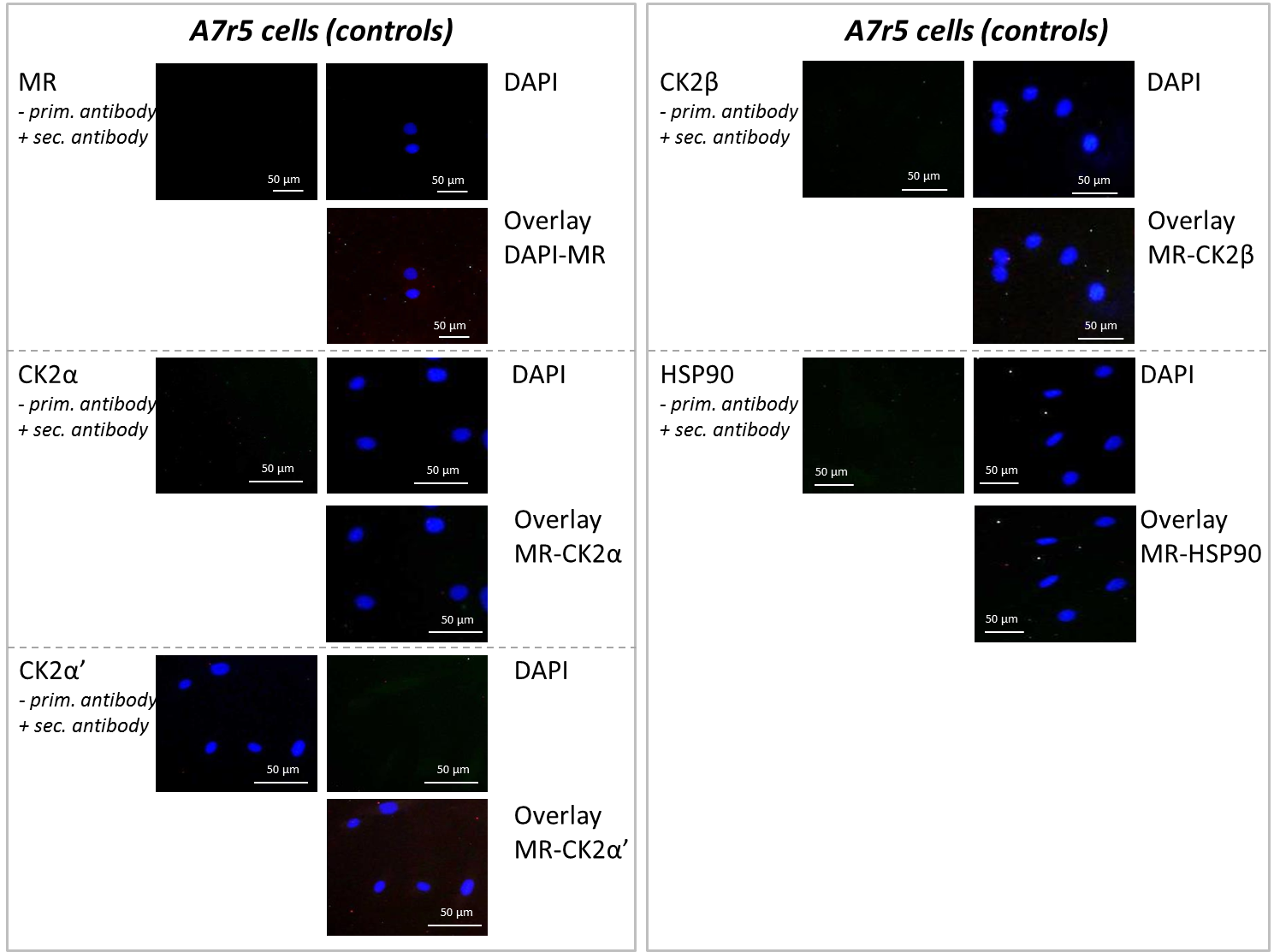
**

**Figure S8B-C: Presentation of the negative controls belonging to the immunofluorescence (IF) analysis.**

RFR-MR-transfected HEK **(B)** and endogenous MR-expressing A7r5 cells **(C)** were seeded on cover slips. IF analyses were performed as described in the experimental procedure without using the respective primary antibody to determine the exact fluorescence exposure time and to avoid false positive results.

**D)**

**
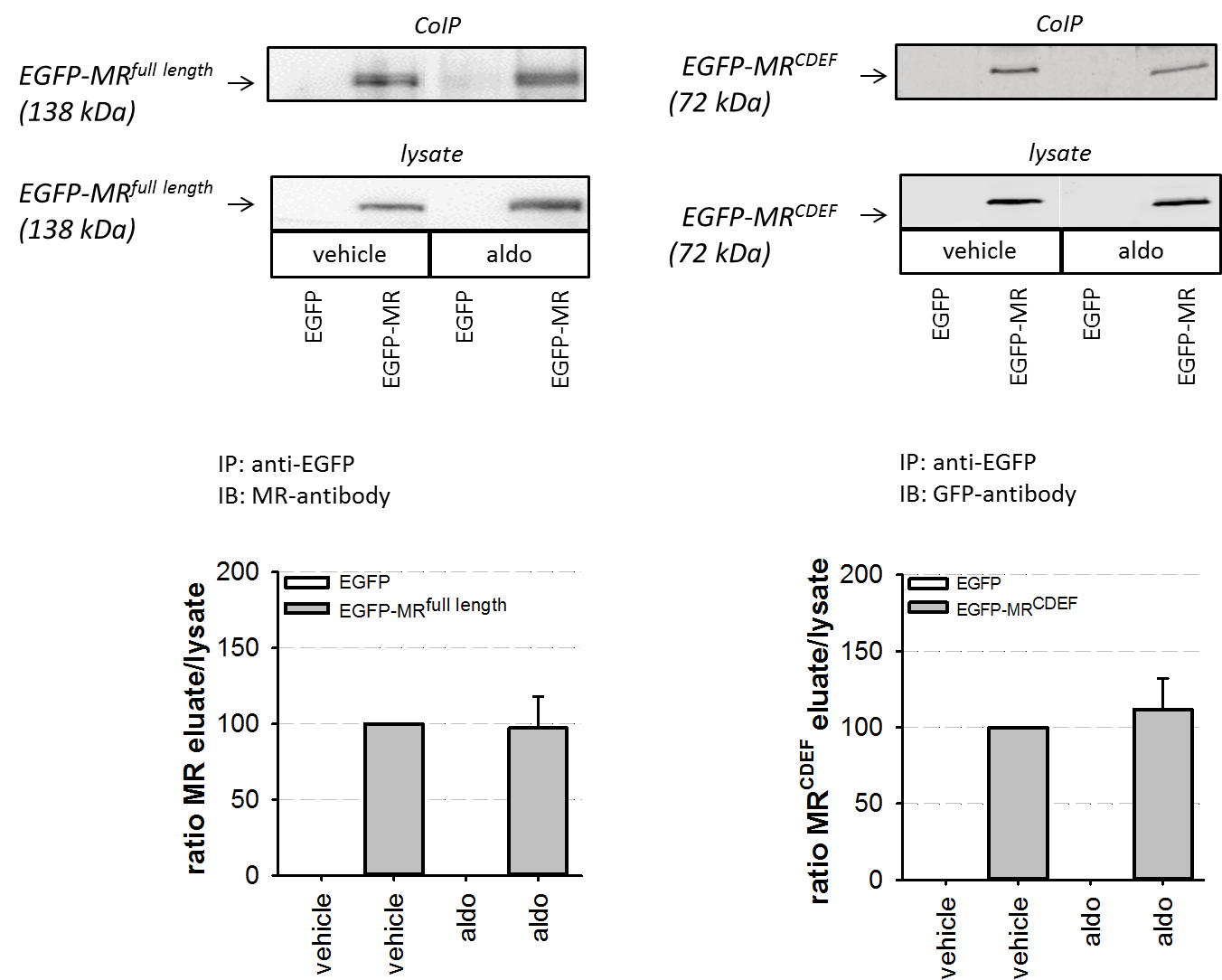
**

**E)**

**
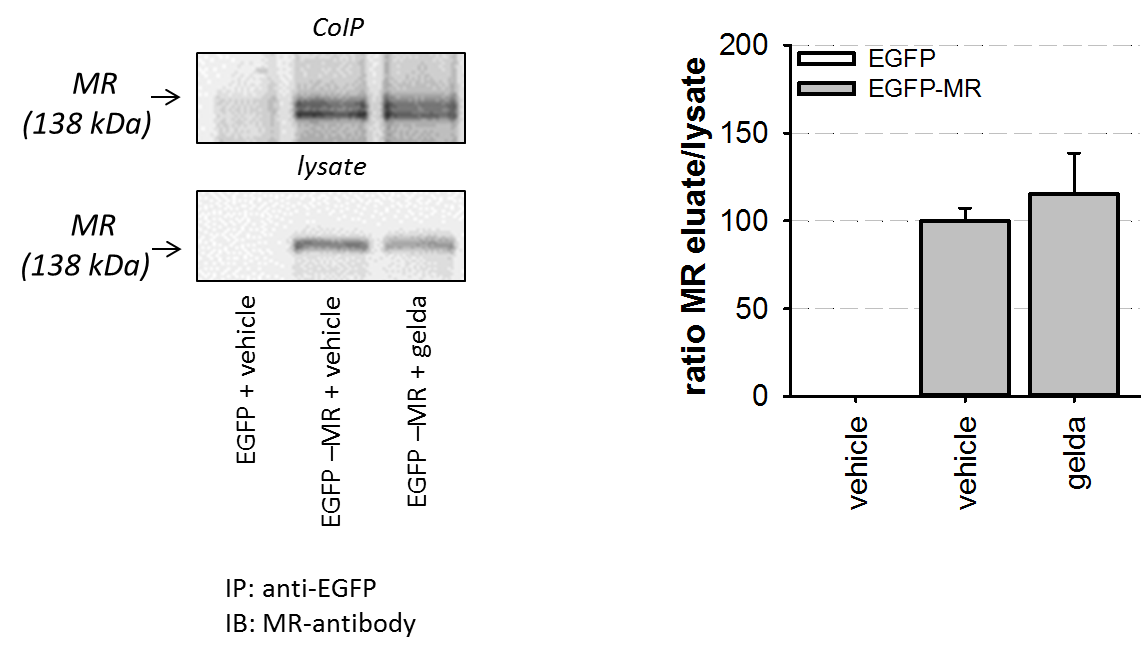
**

**Figure S8D-E: Influence of short termed (D) aldosterone and (E) geldanamycin treatment on the MR expression levels in the cell lysates and CoIP eluates.**

**(D)** CoIP experiments of EGFP-, EGFP-MR and EGFP-MR^CDEF^ transfected HEK cells followed by EGFP-MR pulldown and MR immunoblotting were performed with whole cell lysate after incubating the cells with vehicle (DMSO 0.1%) or aldosterone (10 nM) for 1h. The ratio between the respective MR content of CoIP eluate and the corresponding MR content in the cell lysate was calculated and show no differences concerning the MR ratio of vehicle and aldosterone stimulated cells for both receptor variants (n=2-7; N=2-7).

**(E)** CoIP experiments of EGFP- and EGFP-MR transfected HEK cells followed by EGFP-MR pulldown and MR immunoblotting were performed with whole cell lysate after incubating the cells with vehicle (DMSO 0.1%) or geldanamycin (2 µM) for 2 h. The ratio between the respective MR content of CoIP eluate and the corresponding MR content in the cell lysate was calculated and show no difference concerning the MR ratio of vehicle and geldanamycin stimulated cells (n=8; N=3)**.**

**Figure S9, related to Figure 5C**

**A)**

**
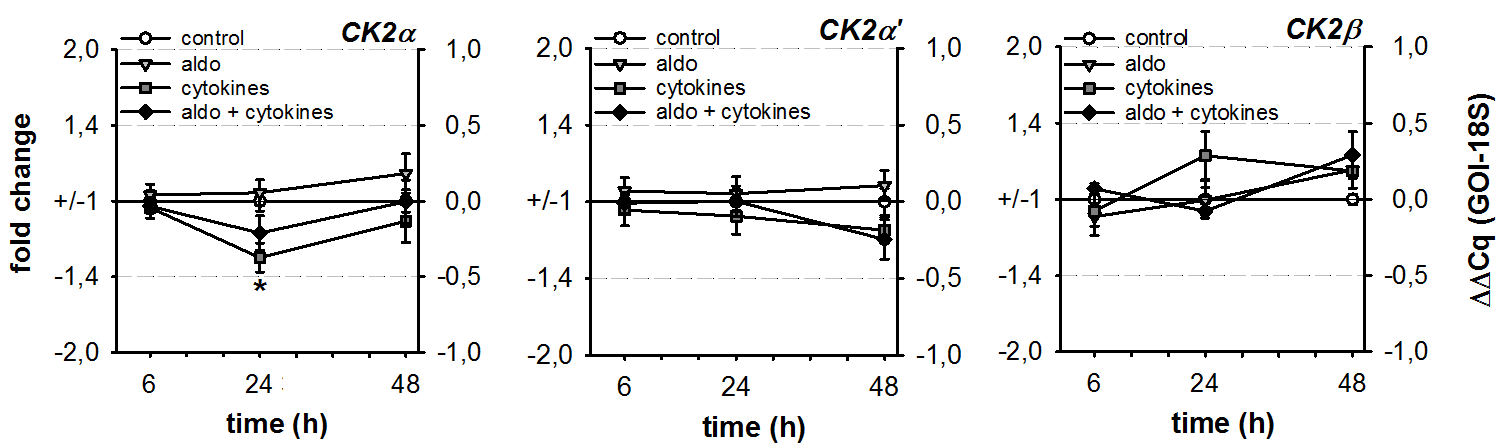
**

**B)**

**
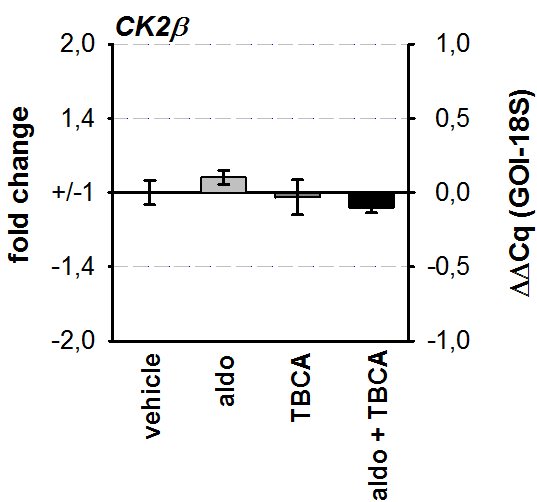
**

**Figure S9:** **Influence of (A) aldosterone and cytokine exposure and (B) TBCA on the mRNA expression of the respective CK2 subunits.**

**(A)** MR-transfected HEK cells were treated with vehicle, aldosterone, a cytokine mixture composed of IL-1β (10 ng/ml), IL-6 (20 ng/ml) and TNF-α (20 ng/ml) or a combination of both for the indicated time points. The mRNA expression of CK2α, CK2α’ and CK2β is presented.

**(B)** MR-transfected HEK cells were treated with vehicle, aldosterone, TBCA (25 µM) or a combination of both for 24 h. The mRNA expression of CK2β is presented.

**Figure S10, related to Figure 5E**

**Figure S10: Impact of the MR on aldosterone-induced NFκB activation.**

NFκB-SEAP reporter gene assays were conducted utilizing MR-transfected HEK cells. The cells were treated with vehicle, aldosterone (10 nM) ± eplerenone (10 µM; MR antagonist) for 48 h and indicate that the aldosterone-induced NFκB activation is mediated by MR dependent mechanisms (n = 6; N = 2; *p ≤ 0.05 vs. control; #p ≤ 0.05 as indicated).

**Figure S11, related to table 1**

**A)**

**
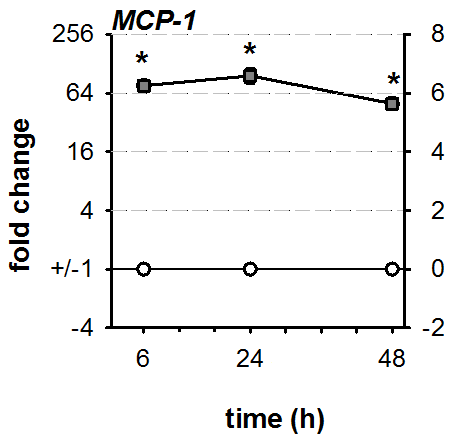

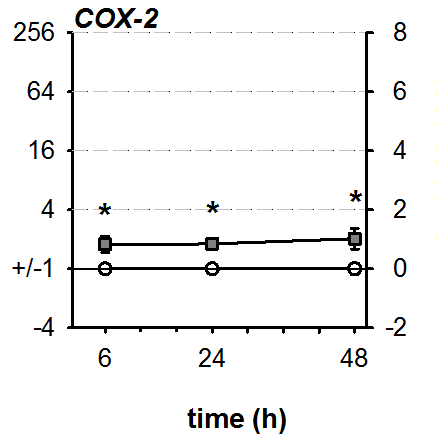

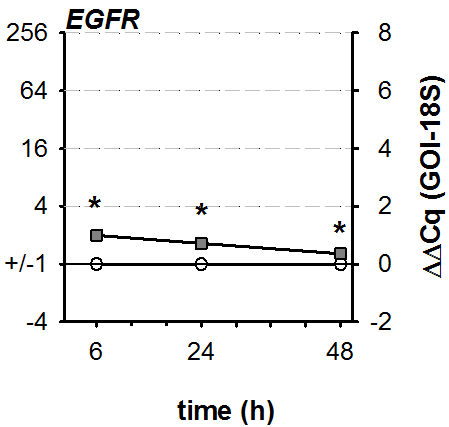
**

**
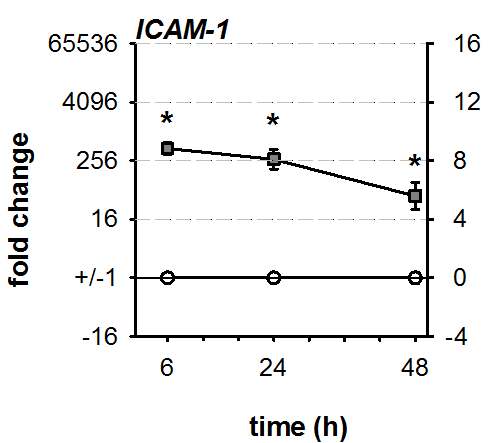

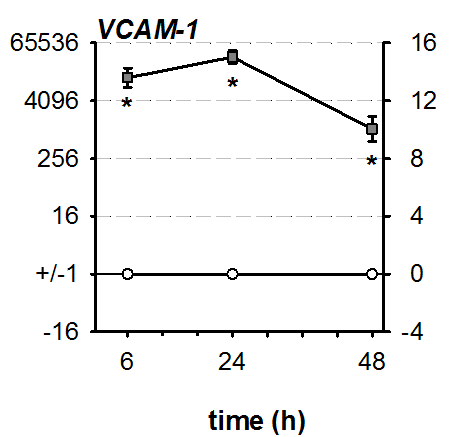

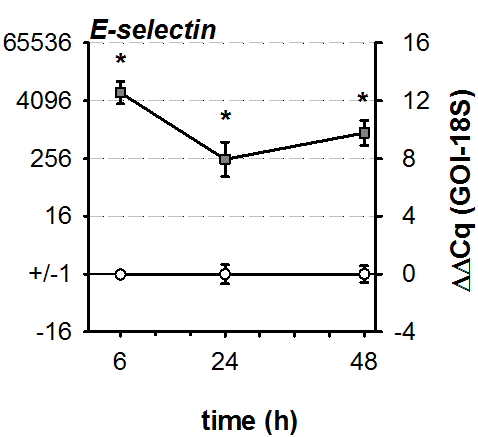
**

**
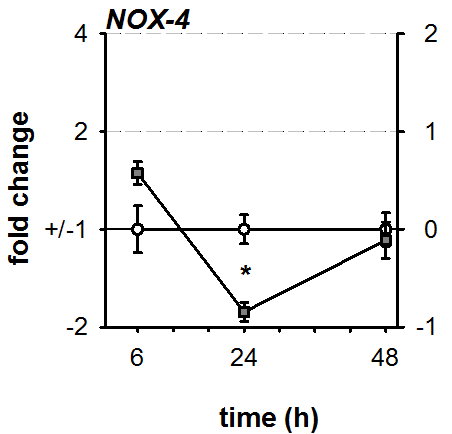

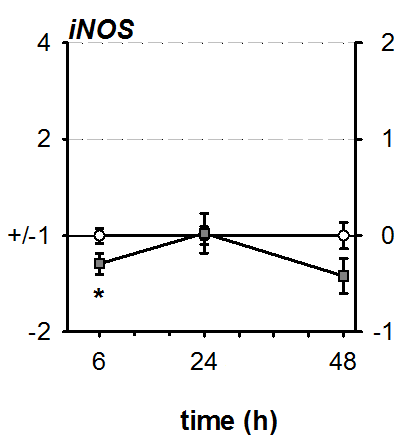
**

**B)**

**
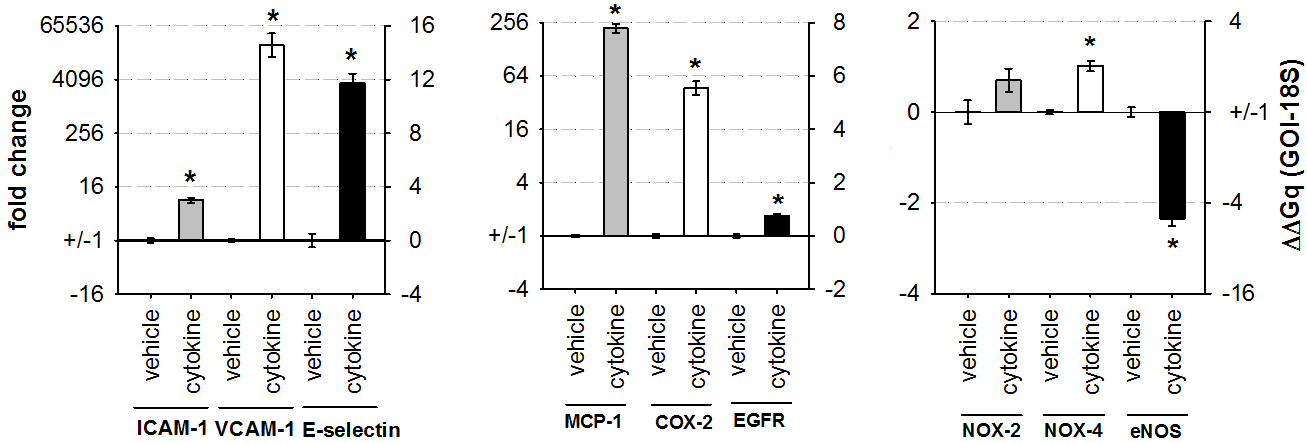
**

**Figure S11:** **Cytokine-induced upregulation of inflammation-associated genes.**

**(A)** MR-transfected HEK cells were treated with vehicle or a cytokine mixture composed of IL-1β (10 ng/ml), IL-6 (20 ng/ml) and TNF-α (20 ng/ml) for the indicated time points. Cytokine-induced mRNA expression of MCP-1, COX-2, EGFR, ICAM-1, VCAM-1, E-selectin, NOX-4 and iNOS is presented. Cytokine-induced fold change and ΔΔCq values at 6, 24, 48 h are shown (n=9-12; N=3-4;*p ≤ 0.05 cytokine vs. cytokine + aldosterone).

**(B)** Cytokine-induced mRNA expression of MCP-1, COX-2, EGFR, ICAM-1, VCAM-1, E-selectin, NOX-4 and eNOS is shown after treatment of endothelial (TIME) cells with vehicle or a cytokine mixture composed of IL-1β (10 ng/ml), IL-6 (20 ng/ml) and TNF-α (20 ng/ml) for 24 h (n=12; N=4; *p ≤ 0.05 cytokine vs. cytokine + aldosterone).

**Original Western Blots:**

**Original Western Blots 1 relating to Figure 1E**:

Original unedited Western blot images of Figure 1E. Framed regions indicated the utilized regions for Figure 1E. Western blot membranes were incubated with anti-MR antibody and reprobed with anti-HSP90 or anti-GAPDH antibody as loading control.


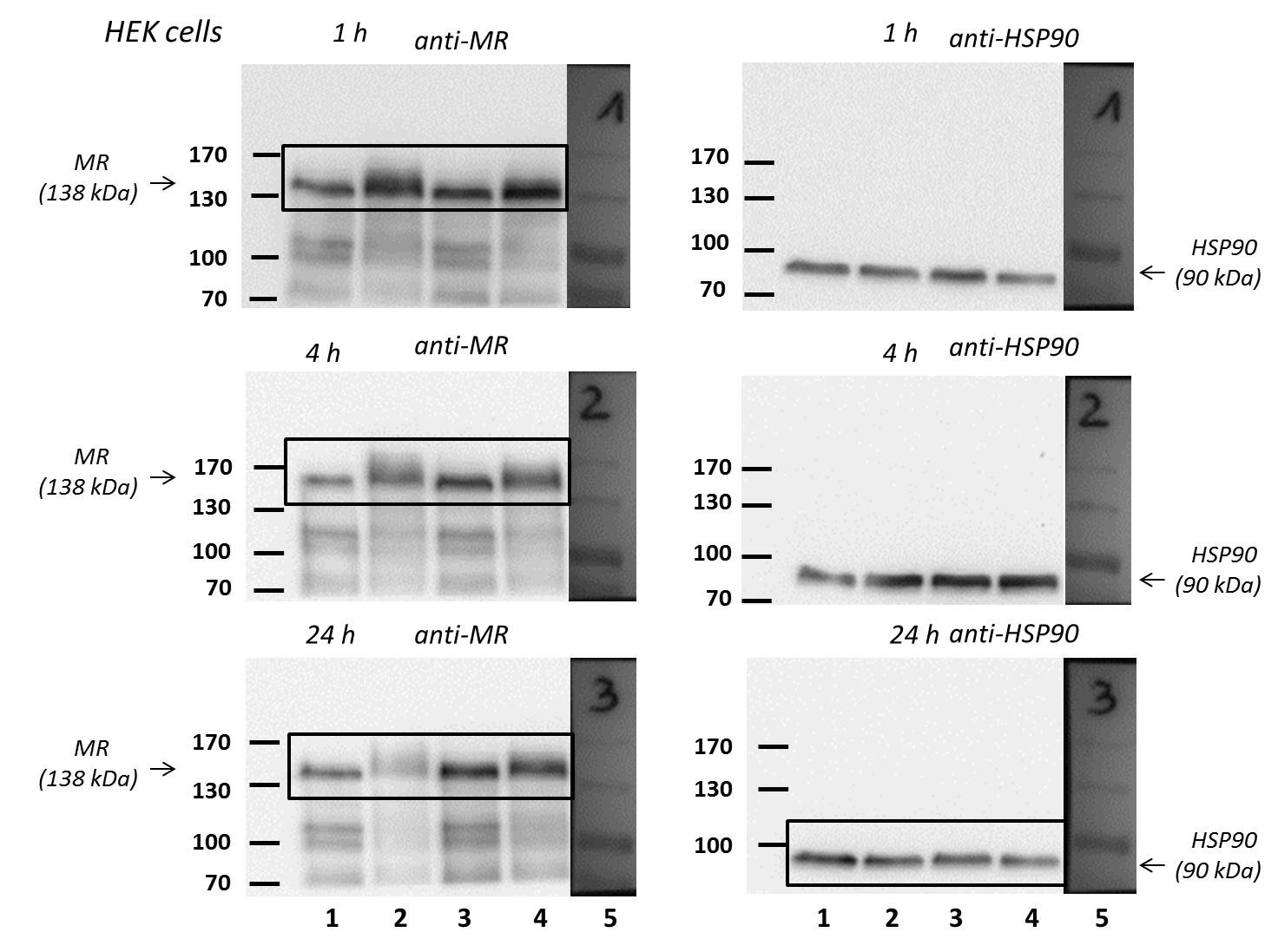


1: vehicle;

2: aldo;

3: TBCA;

4: aldo +TBCA

5: marker


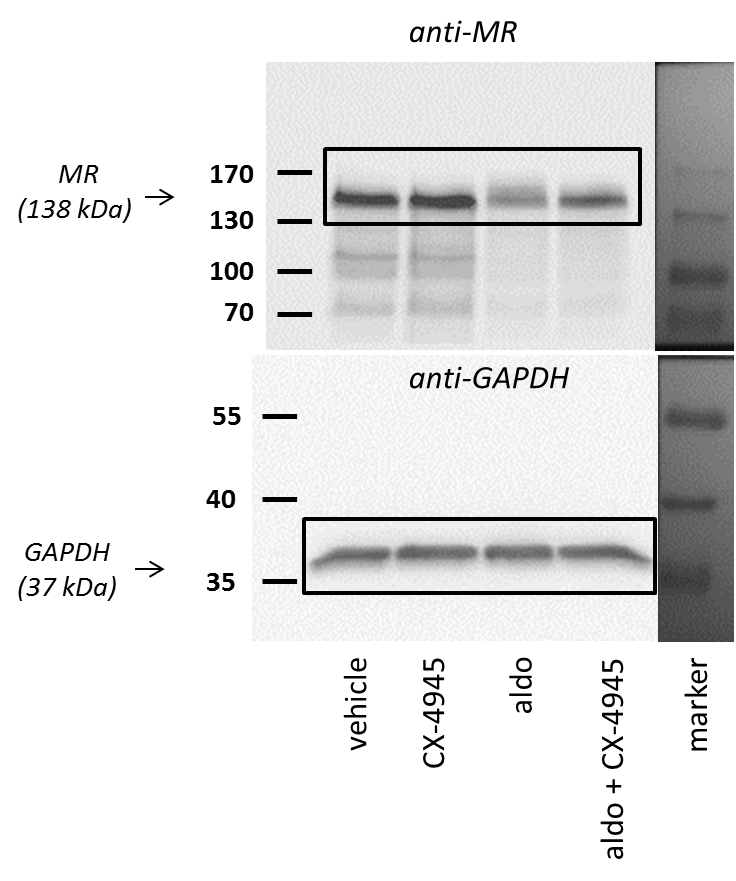


**Original Western Blots 2 relating to Figure 1F:**

Original unedited Western blot images of Figure 1F. Framed regions indicated the utilized regions for Figure 1F. Western blot membranes were incubated with anti-MR antibody and reprobed with anti-HSP90 or GAPDH antibody as loading control.


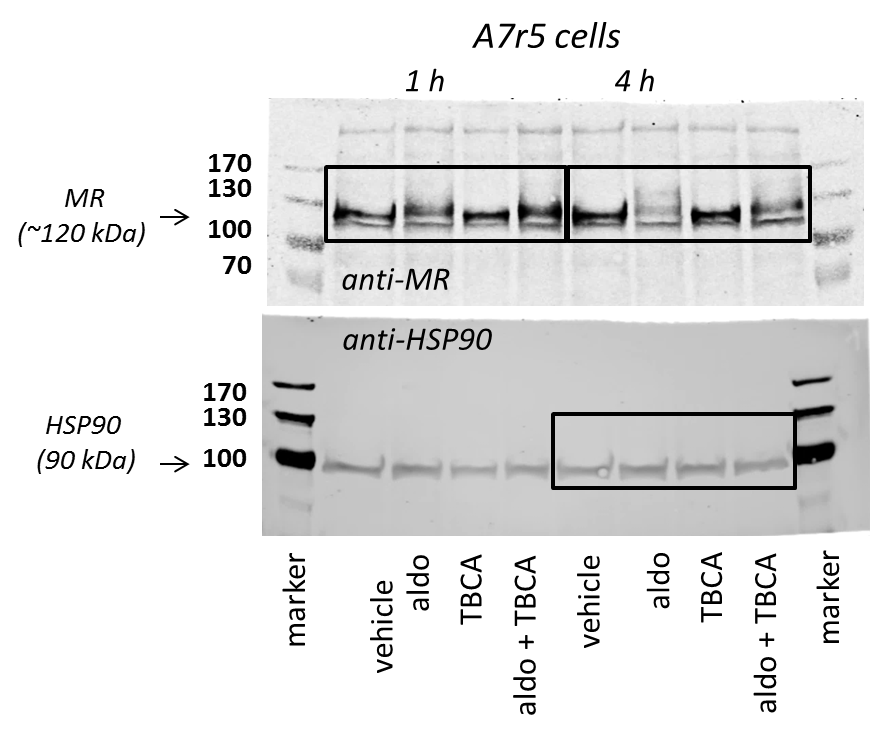


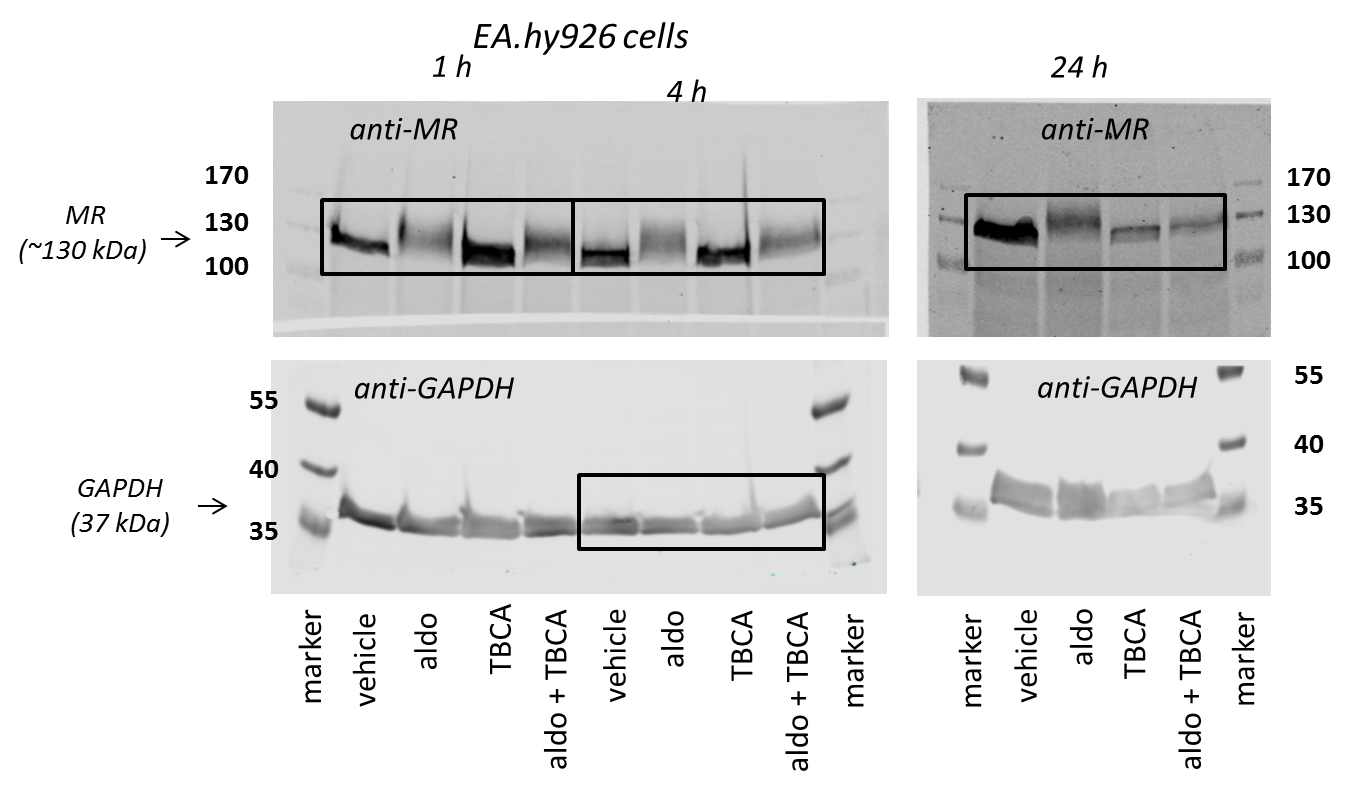


**Original Western Blots 3 relating to Figure 3C**:

Original unedited Western blot images of Figure 3C. Framed regions indicated the utilized regions for Figure 3C. Western blot membranes were incubated with anti-MR antibody and reprobed with anti-HSP90 antibody as loading control.


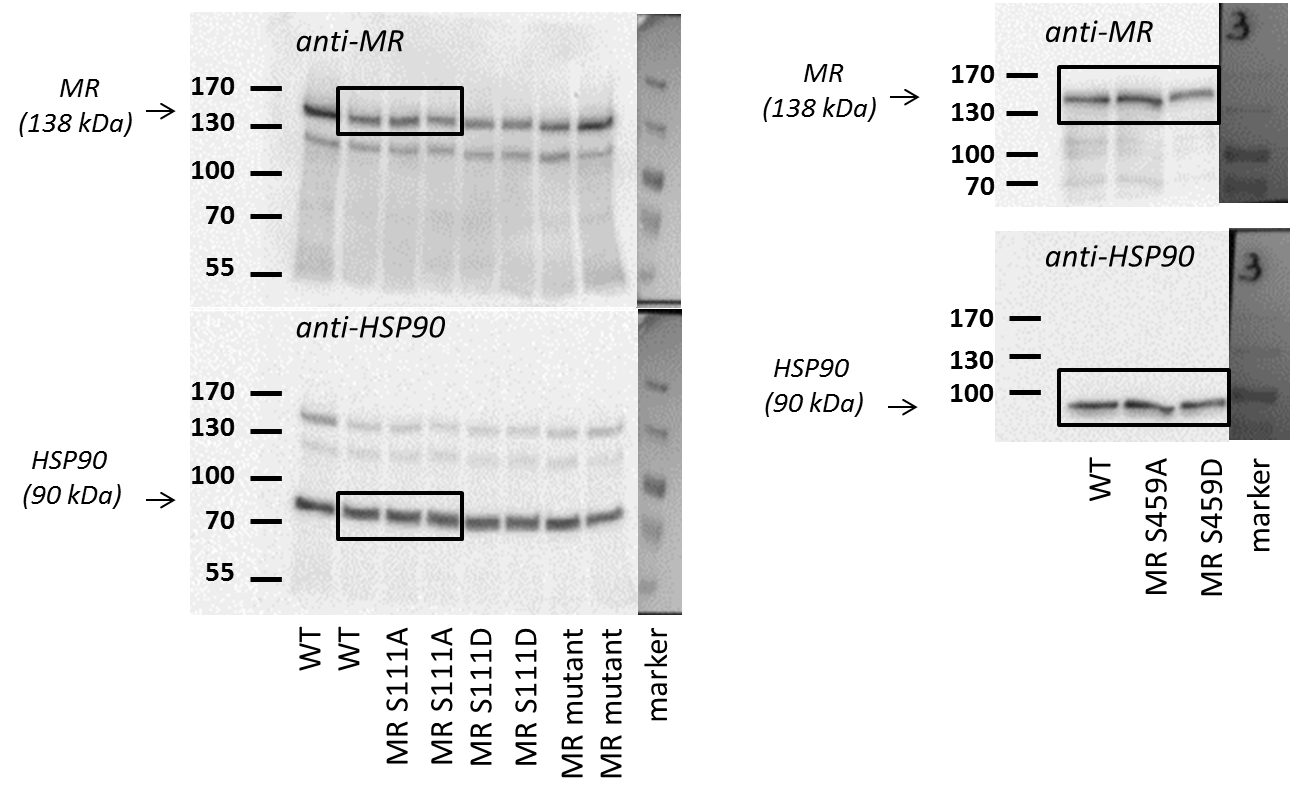


**Original Western Blots 4 relating to Figure 4A**:

Original unedited Western blot images of Figure 4A. Framed regions indicated the utilized regions for Figure 4A. Western blot membranes were incubated with anti-CK2α, anti-CK2α’and anti-CK2β antibody.


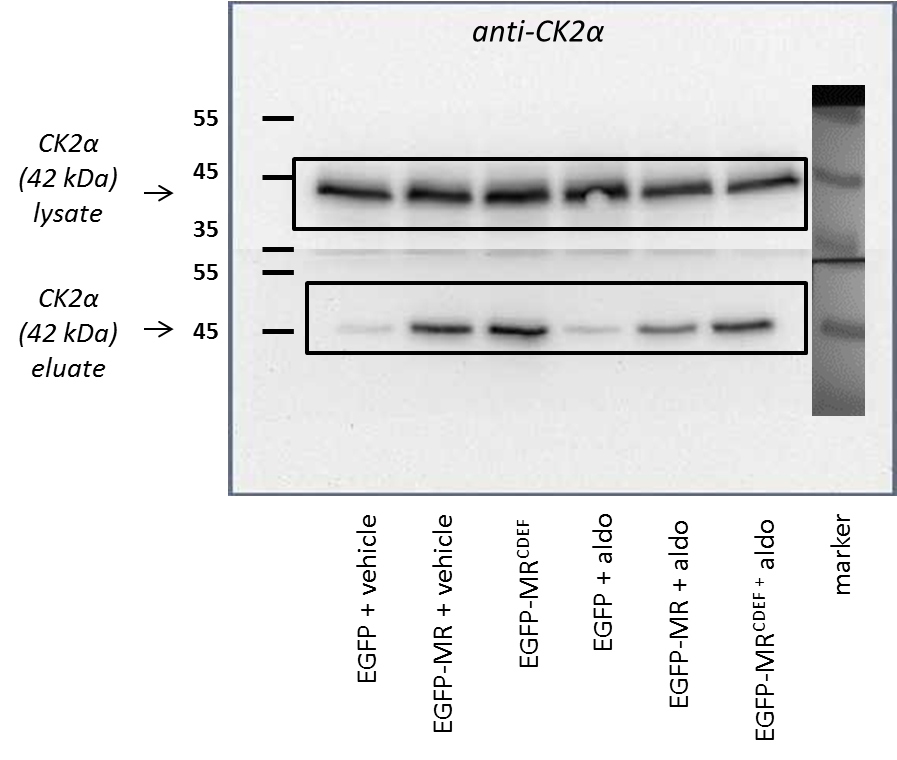


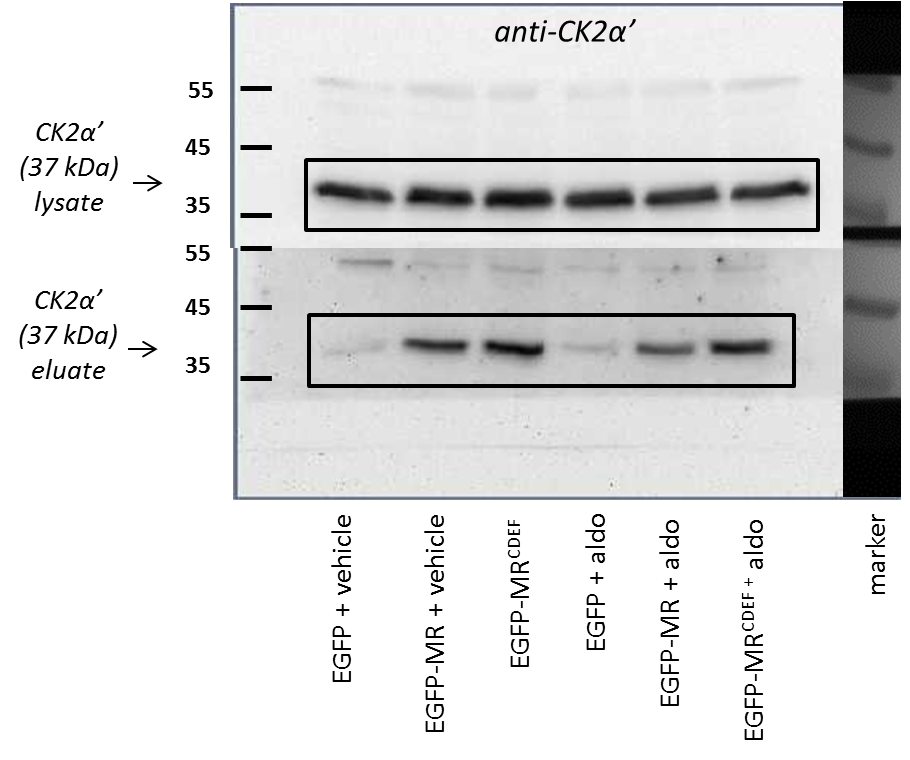


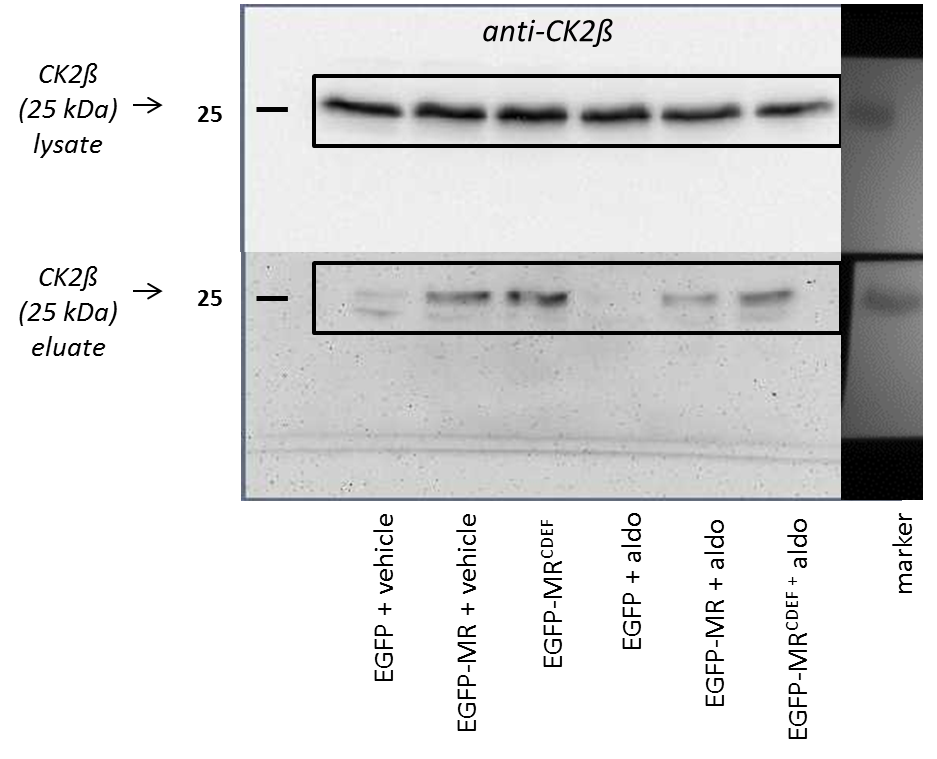


**Original Western Blots 5 relating to Figure 4D**:

Original unedited Western blot images of Figure 4D. Framed regions indicated the utilized regions for Figure 4D. Western blot membranes were incubated with anti-CK2α, anti-CK2α’and anti-CK2β antibody.

***Original Western blots 5 = Original Western blots corresponding to Figure 4D:***


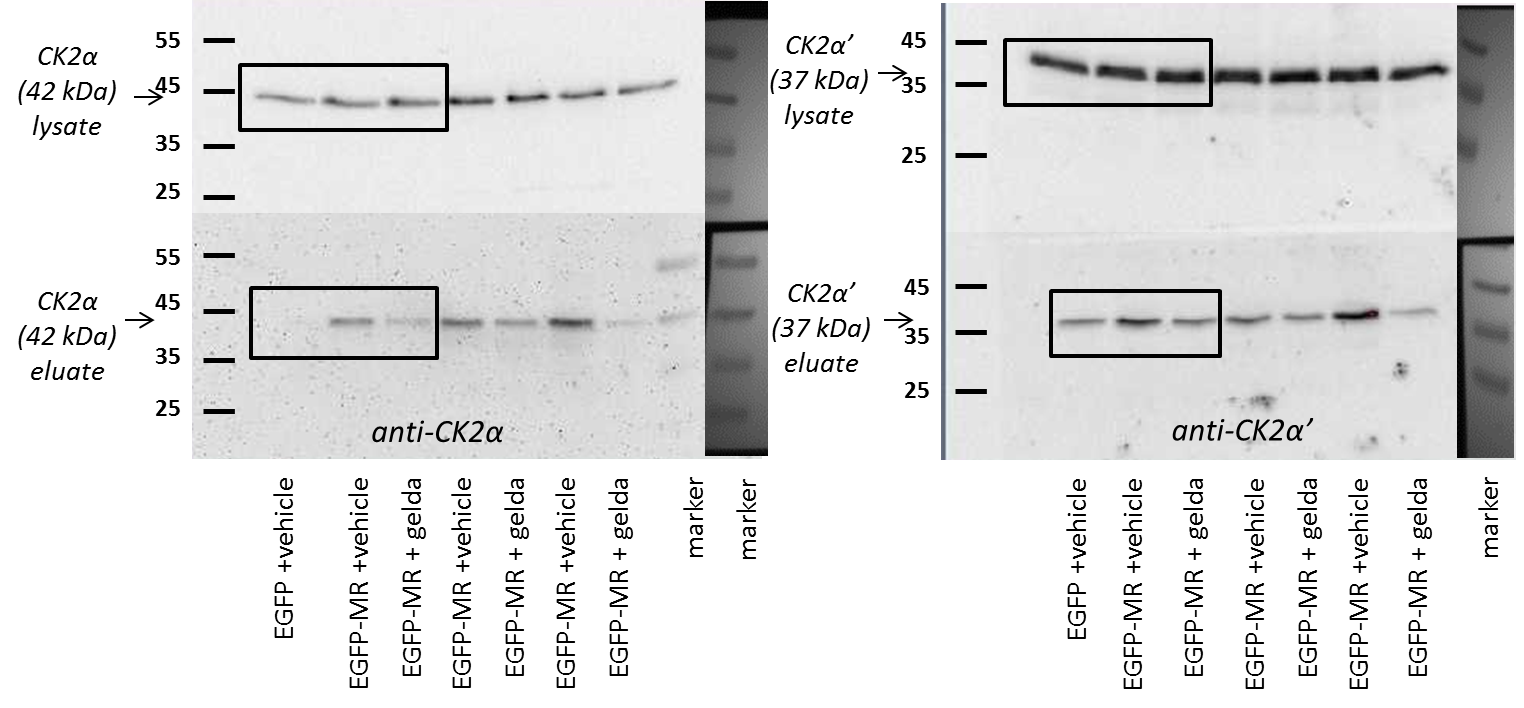


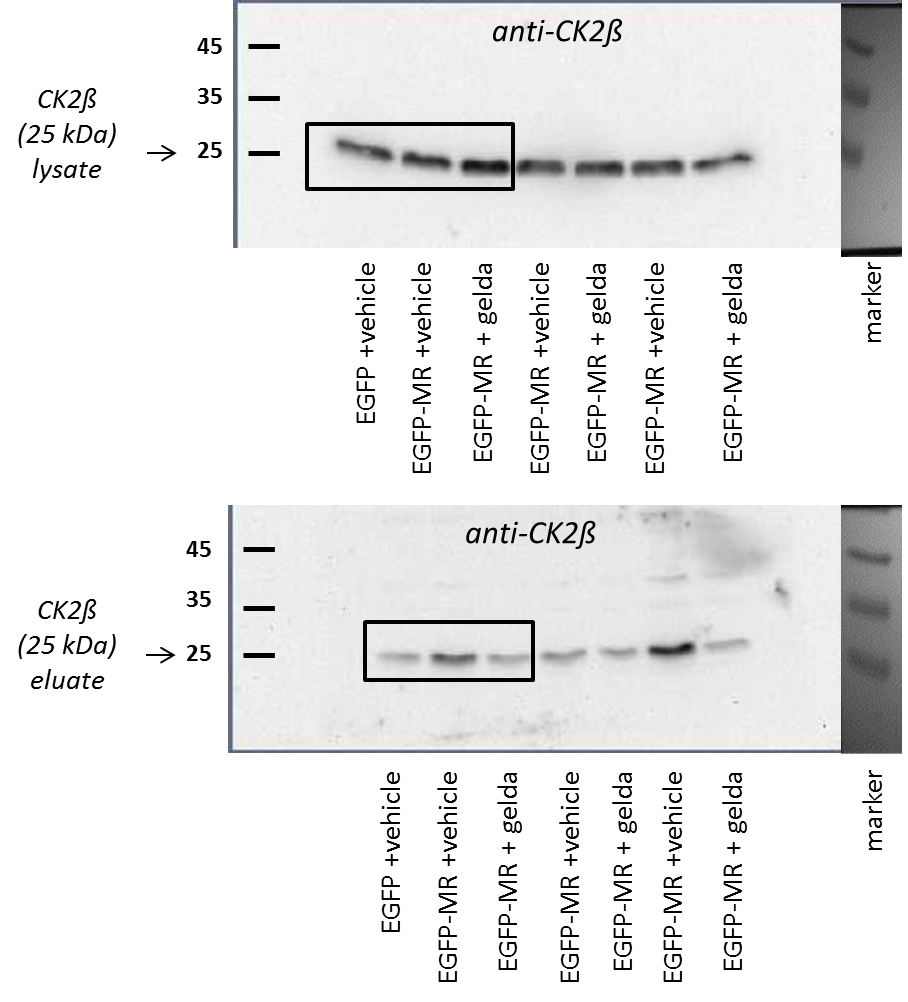


**Original Western Blots 6 relating to Figure 5B**:

Original unedited Western blot images of Figure 5B. Framed regions indicated the utilized regions for Figure 5B. Western blot membranes were incubated with anti-MR, anti-CK2α, anti-CK2α’, anti-CK2β antibody and reprobed with anti-HSP90 antibody as loading control.


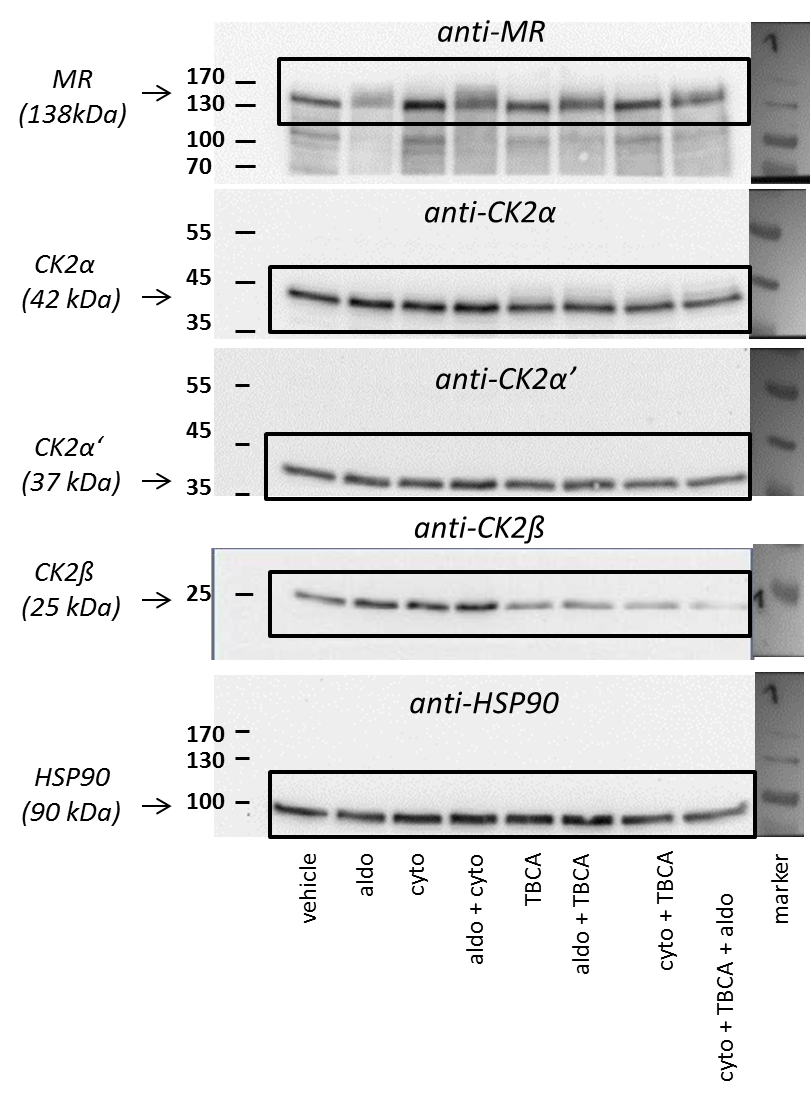


**Original Western Blots 7 relating to supplemental Figure S6.**

Original unedited Western blot images of supplemental Figure S6. Framed regions indicated the utilized regions for supplemental Figure S6. Western blot membranes were incubated with anti-MR antibody and and reprobed with anti-HSP90 antibody as loading control.


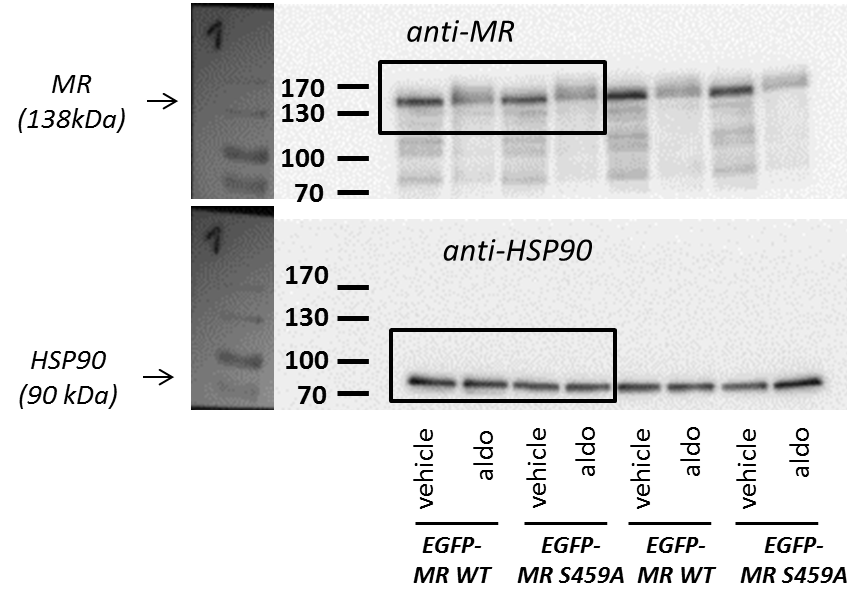


**Original Western Blots 8 relating to supplemental Figure S8A.**

Original unedited Western blot images of supplemental Figure S8A. Framed regions indicated the utilized regions for supplemental Figure S8A. Western blot membranes were incubated with anti-GFP antibody and and reprobed with anti-HSP90 antibody as loading control.


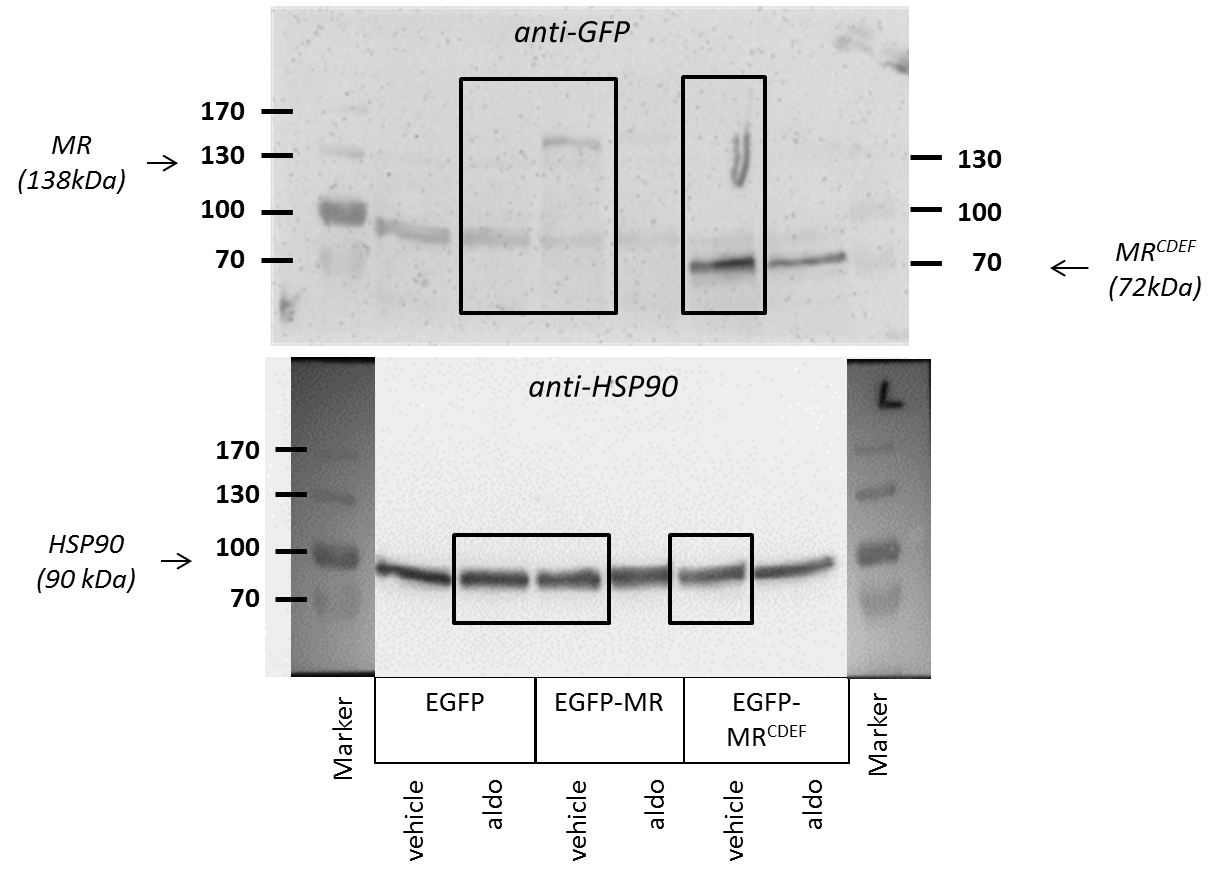


**Original Western Blots 9 relating to supplemental Figure S8D**

Original unedited Western blot images of supplemental Figure S8D. Framed regions indicated the utilized regions for supplemental Figure S8D. Western blot membranes were incubated with anti-MR and anti-GFP antibody for detection.


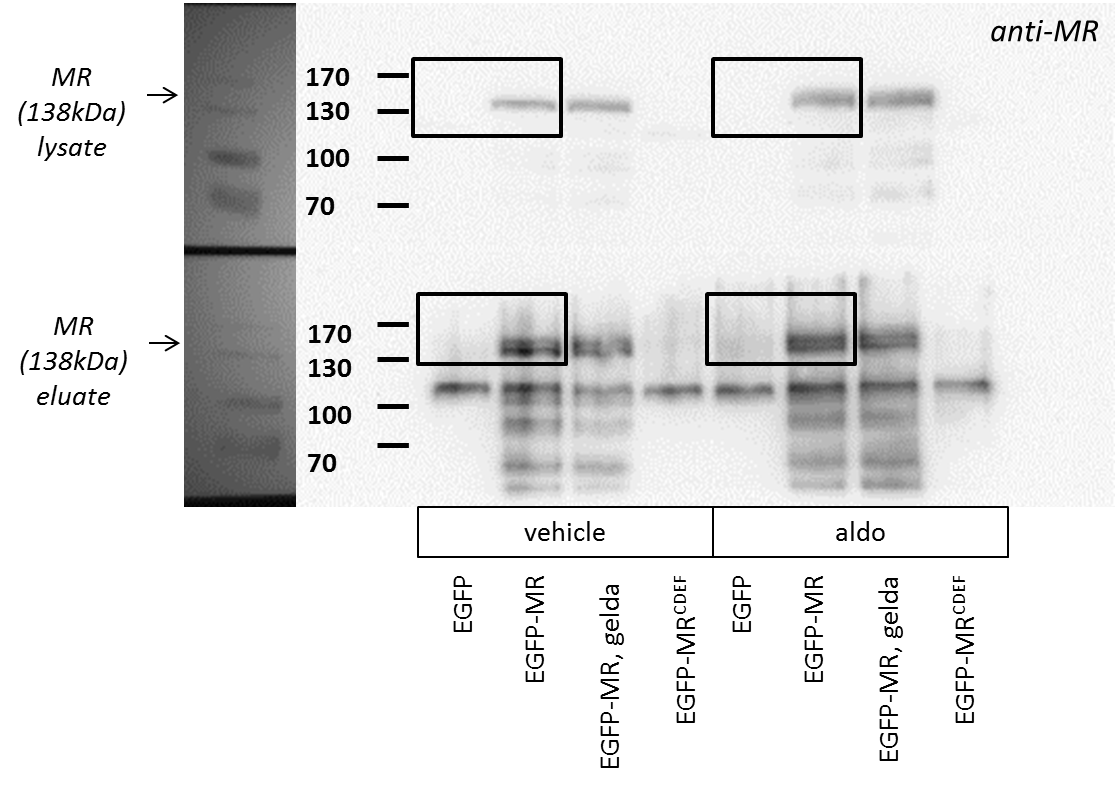


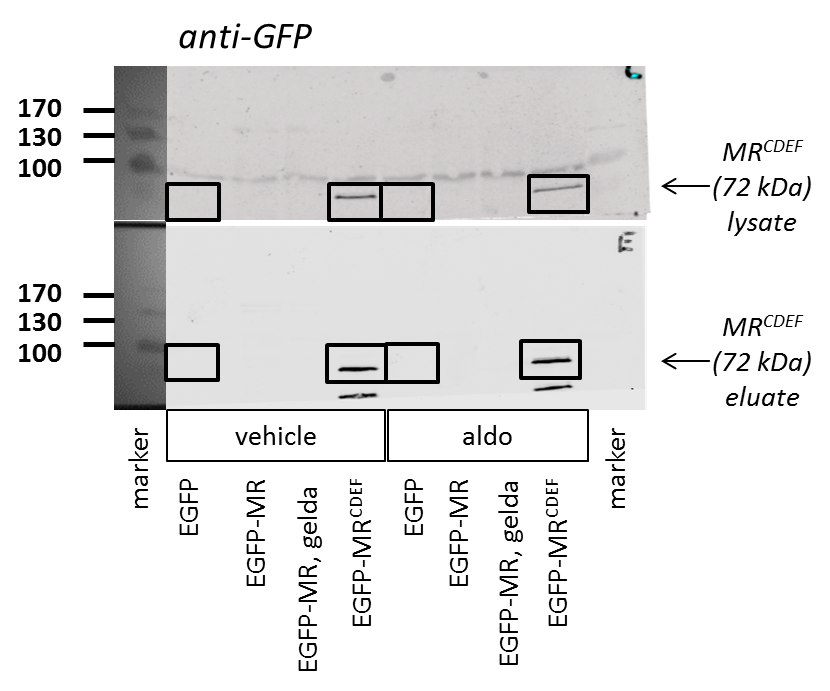


**Original Western Blots 10 relating to supplemental Figure S8E.**

Original unedited Western blot images of supplemental Figure S8E. Framed regions indicated the utilized regions for supplemental Figure S8E. Western blot membranes were incubated with anti-MR antibody for detection.


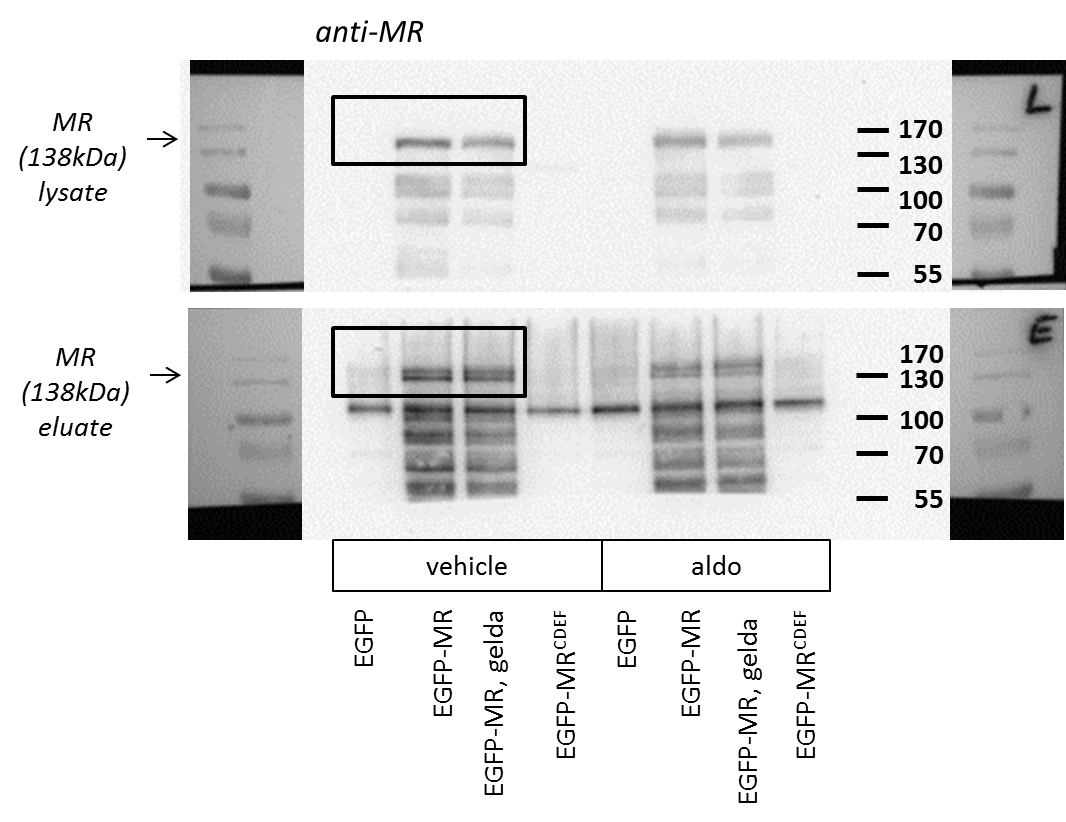


**Supplemental Experimental Procedures**

**Table S2, related to Experimental Procedures (Cell Culture)**

| **Cell line** |  | **Medium composition** |
| --- | --- | --- |
| **HEK-293** | Human embryonic kidney 293 cells | DMEM/Ham’s F-12, 10% FCS |
| **A7r5** | Smooth muscle-derived cell line | DMEM, 10% FCS |
| **EA.hy926** | Human endothelial cell line | DMEM, 10% FCS |
| **TIMEs** | Telomerase immortalized human microvascular endothelial cells | Basal microvascular endothelial cell growth medium, 5% FCS,  in ng/ml: VEGF (5), EGF (5), FGF basic (5), IGF (15),  L-glutamine (10mM), heparin sulfate (0.75 U/ml),  Hydrocortisone (1 µg/ml) , ascorbic acid (50 µg/ml) |

**Table S2: Media composition for cell culture.**

**Table S3A, related to Experimental Procedures (Western Blot)**

| **Chemical product** |
| --- |
| NaCl 150 mM |
| Tris (Base) 10 mM; pH 7.4 |
| Na-orthovanadate 1mM |
| EDTA 1mM |
| Triton X-100 0.1% |
| Nonidet P-40 1% |
| SDS 0.1% |
| Na-deoxycholate 1% |
| protease inhibitor cocktail |

**Table S3A: RIPA Lysis buffer composition.**

**Table S3B, related to Experimental Procedures (Western Blot)**

| **primary antibody** | **Dilution** | **company** |
| --- | --- | --- |
| anti-rMR1-18 1D5 | 1:1000 | DSHB, University of Iowa, USA |
| anti-CK2α | 1:1000 | Cell Signaling, USA |
| anti-CK2α’ | 1:500 | Santa Cruz Biotechnologies, USA |
| anti-CK2β | 1:500 | abcam, UK |
| anti-β-actin | 1:3000 | Cell Signaling, USA |
| anti-HSP90 | 1:3000 | Cell Signaling, USA |
| Anti-GFP | 1:1000 | Cell Signaling, USA |
| **secondary antibody** |  |  |
| anti-mouse IgG HRP-coupled | 1:3000 | Cell Signaling, USA |
| anti-rabbit IgG HRP-coupled | 1:3000 | Cell Signaling, USA |
| anti-goat IgG HRP-coupled | 1:5000 | Santa Cruz Biotechnologies, USA |
| anti-rabbit IgG HRP-coupled | 1:3000 | Cell Signaling, USA |
| anti-rabbit IgG HRP-coupled | 1:3000 | Cell Signaling, USA |
| anti-rabbit IgG HRP-coupled | 1:3000 | Cell Signaling, USA |

**Table S3B: Antibodies used for Western blot analysis.**

Table S4, related to Experimental Procedures (Immunofluorescence analysis)

| **primary antibody** | **Dilution** | **company** |
| --- | --- | --- |
| anti-CK2α | 1:100 | Cell Signaling, USA |
| anti-CK2α’ | 1:100 | Santa Cruz Biotechnologies, USA |
| anti-CK2β | 1:50 | abcam, UK |
| anti-rMR1-18 1D5 | 1:50 | DSHB, University of Iowa, USA |
| anti-HSP90 | 1:50 | Cell Signaling, USA |

| secondary antibody | Dilution | company |
| --- | --- | --- |
| anti-goat Alexa-Fluor 488 | 1:1000 | Cell Signaling, USA |
| Anti-rabbit Oregon green | 1:1000 | Santa Cruz Biotechnologies, USA |
| Anti-mouse Alexa-Fluor 594 | 1:1000 | abcam, UK |

**Table S4: Antibodies used for immunofluorescent analysis.**

**Table S5, related to Experimental Procedures (Quantitative PCR)**

| **gene** | **sense primer** | **antisense primer** | **annealing temperature [°C]** | **product**  **size [bp]** |
| --- | --- | --- | --- | --- |
| 18S | CTGAGAAACGGCTACCACATC | CCCAAGATCCAACTACGAGC | 57 | 251 |
| ICAM-1 | GCTAGCGCTATAAAGGATCACG | AGGGCAGTTTGAATAGCACATT | 59 | 369 |
| VCAM-1 | CATGTAGTGTCATGGGCTGTGA | CCACCACTCATCTCGATTTCTG | 61 | 241 |
| E-Selectin | TCAGCTGTGATAGGGGTTACCT | AGGTGAAGTTGCAGGATGATTT | 59 | 381 |
| MCP-1 | CTCATAGCAGCCACCTTCATTC | ATCACAGCTTCTTTGGGACACT | 60 | 161 |
| COX-2 | CTTACAATGCTGACTATGGCTAC | AAACTGATGCGTGAAGTGCTG | 58 | 242 |
| EGFR | GGGCTCTGGAGGAAAAGAAA | TCCTCTGGAGGCTGAGAAAA | 54 | 93 |
| NOX-2 | CTGCCACCATGGGGAACTGGG | GCACGCACTGGAACCCCTGAG | 62 | 263 |
| NOX-4 | GACGTCCTCGGTGGAAACTTTTG | GTGACGGTCATCTTGCCACATTC | 62 | 338 |
| eNOS | TCTGCGGCGATGTCACTATGGC | TGCGTATGCGGCTTGTCACCTC | 60 | 186 |
| iNOS | ACAAGCCTACCCCTCCAGAT | TCCCGTCAGTTGGTAGGTTC | 57 | 158 |
| MR | TCTGGGCAGAGCTGGCAGAGGTT | AGCATTGCGGGGAACTCTACCTT | 59 | 140 |
| CK2α | GGATTTCCTGGACAAACTGC | GGGTTGGCACTGAAGAAATC | 52 | 188 |
| CK2α’ | CAATGAGAGAGTGGTTGTAAA | CAGGATCTGGTAGAGTTGCTTA | 56 | 211 |
| CK2β | GAAGCCATGGTGAAGCTCTACT | GTCTTGACTGGGCTCTTGAAGT | 61 | 251 |

**Table S5: Primers used for qPCR analysis.**
